# Nonsurgical Platelet Membrane-Decorated Gold Yarnball Brain Antenna for In Situ Nitric Oxide Generation and Neuronal Regeneration

**DOI:** 10.1021/acsnano.6c12030

**Published:** 2026-09-16

**Authors:** Wan-Chi Pan, Ming-You Shie, Jia-Yue Xu, Thi My Hue Huynh, Hsiu-Ching Liu, Ngoc-Tri Tran, Hoi Man Iao, Hui-Wen Lien, Tsu-Chin Chou, Wen-Hsuan Chiang, Shang-Hsiu Hu

**Affiliations:** † Department of Biomedical Engineering and Environmental Sciences, 34881 National Tsing Hua University, Hsinchu 300044, Taiwan; ‡ Department of Biomedical Engineering, 38019 China Medical University, Taichung 406040, Taiwan; § Xenotransplantation Translational Research Center, 38020 China Medical University Hospital, Taichung 404327, Taiwan; ∥ Department of Bioinformatics and Medical Engineering, Asia University, Taichung 413305, Taiwan; ⊥ National Institute of Cancer Research, National Health Research Institutes, Miaoli County 35053, Taiwan; # Institute of Analytical and Environmental Sciences, National Tsing Hua University, Hsinchu 300044, Taiwan; ∇ Department of Chemical Engineering, 34916 National Chung Hsing University, Taichung 402, Taiwan

**Keywords:** nonsurgical brain antenna, gold yarnballs, nitric oxide, wireless electrical stimulation, neuronal regeneration, traumatic brain injury

## Abstract

Bioelectronic implants for brain stimulation are widely used to treat neurological disorders and promote neuronal repair; however, their clinical application is limited by invasive surgical implantation. To address this challenge, here, platelet membrane-decorated gold yarnballs double as a brain antenna and a miniaturized chemo-electrical reactor for neuron electric stimulus and gas-controlled release in traumatic brain injury (TBI). The platform consists of a nitric oxide (NO) donor-functionalized platelet membrane (PMNO) conjugated onto a gold yarnball nanoparticle (GNP). Upon exposure to an external high-frequency magnetic field (HFMF), the conductive GNP generates eddy currents that electrically stimulate neural regeneration while simultaneously triggering controlled NO release from PMNO. This dual-mode stimulation enhances neurogenesis, suppresses astrocyte activation, and promotes angiogenesis at the injury site, leading to significantly improved synaptic differentiation and neurite outgrowth of neural stem cells (NSCs). *In vivo* studies show that combined wireless electrical stimulation and NO signaling suppress inflammation, enhance vascularization, and promote neuronal regeneration in TBI. Behavioral analyses further reveal improved motor coordination and functional recovery in treated mice. The system offers a noninvasive alternative to implanted bioelectronic devices.

## Graphical abstract

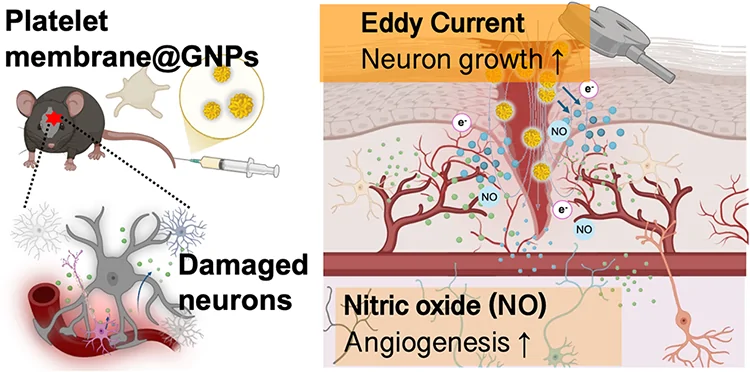

## 1 Introduction

Implantable electronic devices are indispensable tools in biomedical diagnosis and treatment.^1^ In the neuromodulation, bioelectronic implants for brain stimulation provides biological insights and clinical efficacy in neurological diseases.^2^ Despite these advances, implanting electronic devices into the brain still requires invasive intracranial surgery, exposing patients to tissue damage, infection and higher morbidity.^3,4^ To reduce surgical trauma, intravascular electrode systems were developed to avoid direct intracranial puncture.^5^ However, these methods still rely on complex vascular interventions, limited spatial coverage and insufficient targeting precision, leaving large areas of the brain unreachable.^6^ Alternative strategies of intravenous stimulants have been explored, but these strategies typically result in non-specific activation of large brain regions, failing to achieve localized neuromodulation.^7,8^ Meanwhile, mature non-invasive techniques such as transcranial magnetic stimulation and transcranial direct current stimulation, while highly safe, suffer from poor spatiotemporal resolution, limiting their ability to precisely modulate neural circuits.^9,10^

Traumatic brain injury (TBI) is a common neurological disorder caused by external mechanical forces, affecting approximately 69 million people worldwide each year and posing a significant public health burden.^11^ Primary injury directly damages neurons, glial cells, and cerebral blood vessels, triggering a cascade of secondary pathological reactions that disrupt neurological function.^12,13^ Despite advances in molecular biology and rehabilitation, effective clinical interventions remain limited due to secondary injury processes such as persistent oxidative stress and glial scarring, which hinder neural repair.^14^ Persistent activation of stellate and microglia promotes the formation of dense scar tissue, thereby limiting cellular infiltration at the injury site.^15,16^ Furthermore, immune responses to therapeutic materials further exacerbate inflammation and reduce treatment effectiveness.^17,18^ Therefore, these challenges underscore the need to develop therapeutic strategies that can mitigate secondary injury, limit immune activation, and promote neuroregeneration after TBI.^19^

Nitric oxide (NO) is a key endogenous signaling molecule that regulates vascular tone, immune responses, and neural activity.^20,21^ Although NO has distinct concentration-dependent effects,^22^ controlled NO delivery at concentrations below ∼400 nM promotes angiogenesis through the activation of multiple growth factors.^23,24^ NO also modulates inflammatory mediators, including interleukin-1β, histamine, platelet-activating factor, underpinning its therapeutic potential in tissue repair.^25^ Accordingly, a variety of NO-releasing biomaterials have been developed to accelerate wound healing.^26,27^ In the nervous system, NO functions as a neurotransmitter, activating the cGMP pathway to drive neurite outgrowth and synaptic remodeling after injury, thereby supporting neuronal regeneration.^28,29^ Notably, NO-releasing nanoparticles have demonstrated therapeutic efficacy in neurodegenerative disorders of the central nervous system, such as Alzheimer’s disease (AD).^30^ Despite these advances, clinical translation remains hindered by challenges in achieving precise spatiotemporal control of NO delivery, owing to its instability and limited diffusion under physiological conditions.^31^

Inspired by the intrinsic adhesion of platelets to damaged vasculature, platelet membrane (PM)–based biomimetic strategies have been developed for targeted drug delivery in ischemia–reperfusion (I/R) injury.^32^ Owing to the shared vascular disruption and inflammatory cascades between I/R injury and secondary TBI, these strategies are readily translatable to TBI-targeted therapeutics.^33^ Nanoparticles coated with platelet membranes exhibit superior biological performance compared with conventional delivery systems, such as liposomes or PEGylated nanoparticles.^34,35^ This advantage is largely attributed to the preservation of functional membrane proteins, including CD47, which mediates immune evasion by transmitting “don’t-eat-me” signals to macrophages,^36^ as well as integrins such as CD61 that contribute to vascular adhesion and injury-site targeting.^37^ As a result, platelet membrane-coated nanoparticles demonstrate reduced immune clearance and prolonged circulation times *in vivo.* ^38,39^ Besides, platelet membranes retain intrinsic targeting capabilities, allowing them to selectively recognize activated endothelial cells and damaged vascular sites.^40,41^ Their adhesive properties further promote nanoparticle accumulation at injury-associated thrombotic regions, enhancing local therapeutic concentrations.^42^ This combination of immune evasion and vascular targeting positions platelet membrane-coated nanoparticles as an effective and versatile delivery system for TBI therapy.

Electromagnetic field (EMF)–based neuromodulation approaches, including transcranial direct current stimulation (tDCS) and transcranial magnetic stimulation (TMS), have been widely explored for neurological disorders owing to their ability to activate voltage-gated sodium channels and calcium-dependent regenerative signaling.^43,44^ However, the diffuse nature of EMF limits spatiotemporal targeting precision; emerging evidence suggests that electromagnetized gold nanoparticles (GNPs) can localize and amplify EMF-induced neuromodulation,^45,46^ thereby enhancing therapeutic efficacy in central nervous system disorders.^47,48^ Here we developed a brain-electronic antenna (BEA) that, following intravenous injection, circulates through the bloodstream and autonomously localizes at TBI sites. The BEA enables served as brain antenna and miniaturized chemo-electrical reactor for neuron electric stimulus and gas-controlled release at TBI. Realization of the BEA requires overcoming several key hurdles: (i) development of efficient, wireless, free-floating electronic devices miniaturized to fit within damaged vessels; (ii) sustained circulation without rapid clearance from the bloodstream; and (iii) specific recognition of and self-implantation at TBI regions. To address these challenges, we engineered wireless-charging, platelet membrane (PM)–decorated gold yarnball nanoparticles (GNP) functionalized with a nitric oxide (NO) donor (Figure 1a). Under an external high-frequency magnetic field (HFMF), the conductive gold core generates eddy currents for neural electrical stimulation while simultaneously triggering controlled NO release (Figure 1b). This dual-mode stimulation enhances neurogenesis, suppresses astrocyte activation, promotes angiogenesis, and supports neuronal regeneration *in vivo*, underscoring the platform’s potential for targeted neuromodulation. Accordingly, the system could be applied to a range of neurological disorders, including Alzheimer’s disease,^49^ ischemic stroke,^50^ brain tumors,^51^ neuropathic pain,^52^ and spinal cord injury,^53^ where therapies may benefit from precise electrical modulation of inflamed or affected regions.

**Figure 1:**
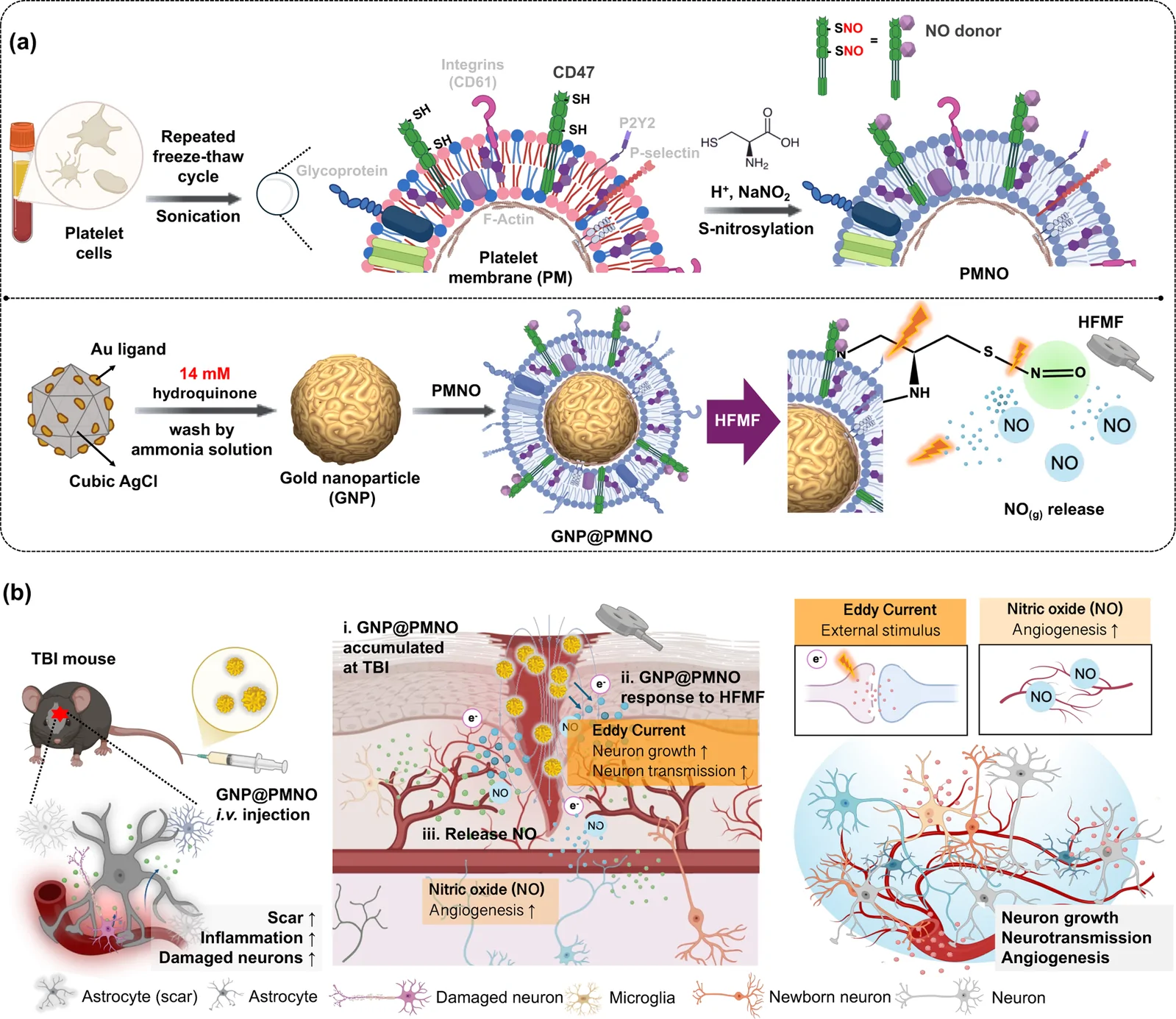
High-frequency magnetic field (HFMF)-responsive NO-releasing gold nanoparticles (GNP@PMNO) for targeted TBI therapy. (a) Schematic illustration of the GNP@PMNO design. Gold nanoparticles (GNPs) are functionalized with platelet membrane proteins containing cysteine residues, allowing conjugation of NO donors via thiol (-SH) groups. Upon exposure to HFMF irradiation, the GNP@PMNO system releases nitric oxide (NO) and generates localized electrical currents. (b) Following intravenous administration, GNP@PMNO enables targeted delivery to the injury site. The HFMF-triggered release of NO and electrons promotes angiogenesis, neurogenesis, and functional recovery in TBI treatment.

## 2 Results and Discussion

### 2.1 Preparation of PMNO

The versatile platelet membrane–nitric oxide–coated GNPs (GNP@PMNO) was produced by self-assemble of platelet membrane-nitric oxide (PMNO) through ultrasonic against ultrapure water, with an optimal concentration of hydroquinone (HQ) yielding uniform GNPs. Briefly, platelet membranes were isolated by sequential freeze–thaw cycles and sonication to mechanically disrupt platelet structure while preserving membrane integrity (Figure 1a).^54^ Repeated freeze–thaw cycles induce membrane rupture via ice crystal formation and subsequent leakage of intracellular contents upon thawing,^55^ whereas sonication further disrupts cellular remnants through cavitation-induced shear forces. Following mechanical disruption, centrifugation was used to separate membrane fractions from soluble cytosolic components. This detergent-free approach preserves native membrane architecture and functional proteins, rendering the isolated platelet membranes suitable for downstream applications such as S-nitrosation and receptor-related studies.

The platelet membrane protein’s cysteine -SH functional groups were then converted into -SNO under acidic conditions through a reaction with NaNO_2_, resulting in the formation of PMNO. Cysteine residues on platelet membrane proteins contain reactive thiol (-SH) groups, which can undergo S-nitrosation to form S-nitrosothiols (-SNO).^56^ In this process, sodium nitrite (NaNO_2_) under acidic conditions acts as the nitrosating agent. Protonation of nitrite generates nitrous acid (HNO_2_), which further decomposes to form the electrophilic nitrosonium ion (NO^+^), the active species responsible for nitrosation. The sulfur atom of the cysteine thiol, acting as a nucleophile, attacks NO^+^, resulting in the formation of a covalent S–N bond and producing the S-nitrosothiol derivative.^57^ This reaction yields platelet membrane S-nitrosothiol (PMNO), a modified protein with -SNO functional groups.

### 2.2 Size Control of GNPs and Synthesis of GNP@PMNO

The core GNPs were prepared using a seed-growth method,^58^ in which chloroauric acid (HAuCl_4_) is used as the precursor, hydroquinone as the reducing agent, and polyvinylpyrrolidone (PVP) as the surfactant. The obtained GNPs further cloaked with fragments of platelets cell membrane by ultra-sonication, yielding GNP@PMNO (Figure 2a). The diameter of GNPs was systematically tuned by varying the concentration of the reducing agent, hydroquinone, from 1 to 56 mM, and the resulting nanostructures were examined by scanning electron microscopy (SEM). Across all conditions, the synthesized GNPs exhibited a characteristic brain-like architecture with grooved surfaces and porous features, which became more pronounced as particle size increased (Figure 2b). A clear inverse relationship was observed between hydroquinone concentration and GNP diameter, with lower concentrations yielding smaller nanoparticles. Among the tested conditions, GNPs produced at 14 mM hydroquinone displayed a uniform size distribution around 160 nm and well-developed surface topology, while avoiding excessive aggregation at higher concentrations and incomplete structural formation at lower concentrations. On the basis of these considerations, 14 mM hydroquinone was selected as the optimal condition for subsequent studies.

**Figure 2:**
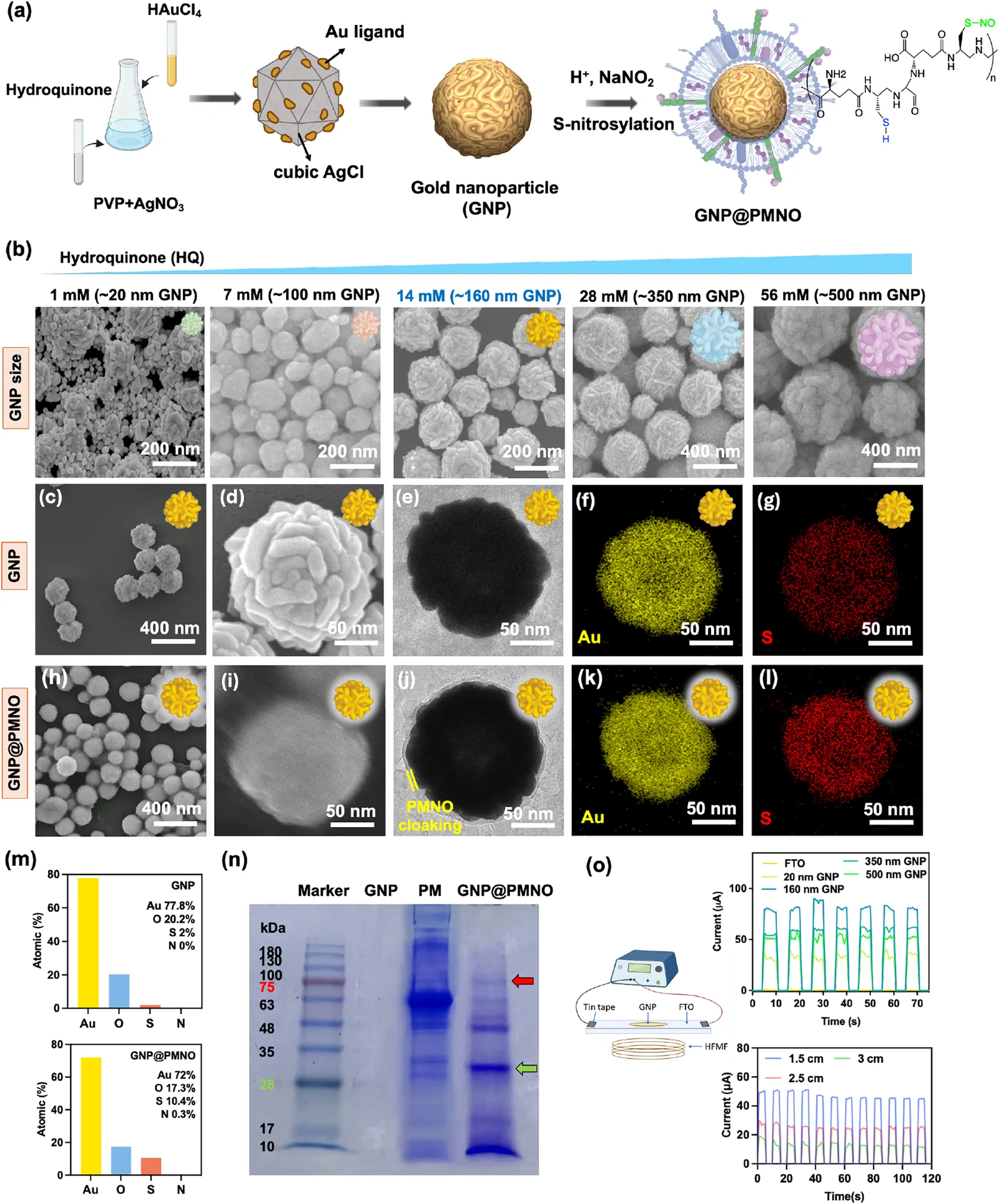
Preparation and characterization of GNP and GNP@PMNO. (a) Schematic illustration of the synthesis of GNP@PMNO. SEM and TEM images, along with elemental mapping, of (b) GNPs with different sizes synthesized using varying hydroquinone (HQ) concentrations, (c–g) GNP, and (h–l) GNP@PMNO. (m) Quantitative analysis of elemental composition derived from EDS mapping. (n) SDS–PAGE analysis of GNP, platelet membrane (PM), and GNP@PMNO. (o) Current–time (I–T) curves of GNPs synthesized with different HQ concentrations (top), demonstrating their electrical response behavior. The effective stimulation intensity is strongly dependent on the distance between the HFMF source and the target system, following electromagnetic field attenuation from the excitation coil (bottom).

The morphology and elemental composition of both bare GNPs and GNP@PMNO, synthesized under the optimized 14 mM hydroquinone condition, were systematically characterized by SEM, transmission electron microscopy (TEM), and energy-dispersive spectroscopy (EDS) (Figure 2c–l). SEM and TEM analyses revealed that bare GNPs were homogeneously dispersed and exhibited a rough, grooved surface topology indicative of controlled nucleation and growth (Figure 2c–e). Following PMNO cloaking, GNP@PMNO retained the overall nanoparticle morphology, while high-magnification images revealed a uniform, semi-transparent shell enveloping the nanoparticle surface, with a thickness of approximately 5–10 nm, consistent with successful membrane coating (Figure 2h–j).

Elemental analysis further corroborated the surface modification. EDS spectra of bare GNPs displayed a dominant gold signal with negligible contributions from heteroatoms, confirming the high purity of the nanoparticles prior to functionalization. In contrast, EDS elemental mapping of GNP@PMNO showed a pronounced sulfur signal, originating from membrane-associated proteins, thereby validating the effective conjugation of PMNO onto the GNP surface (Figure 2f–g,2k–m). To further verify the successful cloaking and structural preservation of membrane-associated proteins, sodium dodecyl sulfate–polyacrylamide gel electrophoresis (SDS-PAGE) analysis was performed (Figure 2n). The overall protein expression profile of GNP@PMNO closely resembled that of native PMNO, indicating that the platelet membrane was successfully translocated onto the GNPs without compromising its native protein composition. To definitively confirm the surface orientation and retention of key functional biomarkers on the biomimetic vectors, we performed dual-parameter flow cytometry analysis targeting the immune-evasive marker CD47 and the platelet-specific integrin CD61 (Figure S1). While bare GNPs exhibited negligible background signals, the membrane-cloaked nanoparticles demonstrated robust surface presentation of both CD47 and CD61, validating the preservation of essential cell-derived functional integrity required for subsequent systemic cloaking and target homing.

### 2.3 Electrical Responsiveness of GNPs and GNP@PMNO under HFMF

The electrical responsiveness of GNPs under HFMF stimulation was evaluated by monitoring current–time (I–T) behavior. GNPs deposited onto fluorine-doped tin oxide (FTO) substrates exhibited clear and reversible current responses that were highly synchronized with periodic HFMF switching (2.8 kW, 1 MHz, 5 s on/off), confirming their capability to generate stable electrical signals upon magnetic stimulation (Figure 2o, top). This reproducible electrical output supports the potential of GNPs as magnetically responsive electrical transducers for subsequent bioelectronic applications.

Notably, the effective stimulation strength is strongly dependent on the spatial distance between the HFMF source and the target system, as the magnetic flux density (B-field) decays with increasing separation distance from the coil according to electromagnetic field attenuation principles.^59^ Since the induced eddy current density in conductive nanostructures is proportional to the time-varying magnetic flux (J ∝ d*B*/d*t*), a reduced local magnetic field at larger distances results in a corresponding decrease in induced current magnitude.^60^ Therefore, the observed electrical response is maximized when the GNP-FTO system is positioned within the near-field region of the HFMF coil, where magnetic field intensity is sufficiently strong and stable to sustain measurable eddy current generation (Figure 2o, bottom).

A size-dependent electrical response was observed, in which larger GNPs generally produced higher current amplitudes under identical HFMF conditions, likely due to their increased specific surface area facilitating interfacial charge accumulation and charge transfer. Notably, GNPs synthesized under the optimized 14 mM hydroquinone condition (160 nm GNPs), which exhibited uniform dispersion and balanced surface morphology, generated robust and stable electrical signals. In contrast, the smaller 20 nm GNPs and ultrasmall GNPs (∼5 nm) exhibited a significantly weaker electrical response, approximately 1.2-fold and 2.8-fold lower than that of the 160 nm GNPs (Figures 2o and S2). This finding suggests that the electrical response is governed not only by particle size but also by the overall structural integrity and interparticle conductive network formation under HFMF stimulation, where the optimized 160 nm GNPs achieve a more favorable balance between surface area and efficient charge transport pathways,^61^ enabling effective interfacial charge accumulation and stable signal transduction,^62^ whereas excessively small nanoparticles may suffer from limited conductive coupling and reduced current propagation efficiency.

To further investigate HFMF-responsive membrane perturbation at the nanoparticle–cell interface, intracellular DAPI penetration was evaluated following HFMF stimulation (Figure S3). Compared with the control group and the corresponding groups without HFMF exposure, significantly enhanced intracellular DAPI fluorescence was observed in both the GNP+HFMF and GNP@PMNO+HFMF groups, indicating increased membrane permeability upon HFMF treatment. Since DAPI is generally impermeable to intact cellular membranes under short incubation conditions, the elevated nuclear fluorescence indicates that conductive nanoparticles subjected to HFMF stimulation induce transient, reversible membrane permeabilization at the nanoparticle–cell interface. Crucially, LIVE/DEAD viability assays confirmed that cell viability remained uncompromised following HFMF exposure (Figure S3), demonstrating that this localized electromagnetic interaction causes temporary, non-lethal membrane destabilization—analogous to reversible electroporation—rather than irreversible structural damage or cytotoxicity. These findings support the proposed mechanism that localized eddy currents generated under HFMF irradiation facilitate temporary membrane permeabilization, enhancing cellular uptake without impairing overall cell survival or functional integrity.

### 2.4 Characteristics of GNP and GNP@PMNO

The surface modification and structural integrity of GNP and GNP@PMNO were systematically characterized using ζ potential analysis, X-ray diffraction (XRD), and high-resolution X-ray photoelectron spectroscopy (XPS). During the reaction series, particle sizes increased from approximately 280 nm to 325 nm (Figure 3a). The larger mean size of GNP@PMNO (∼325 nm) is likely due to the hydrodynamic contribution of PMNO, reflecting its expanded conformation and large gyration radius in aqueous solution. Correspondingly, the ζ potential gradually shifted from −14.37 mV for bare GNPs and −12.43 mV for PMNO to −11.27 mV after PMNO coating, confirming successful surface functionalization (Figure 3b). Thermogravimetric analysis (TGA) further indicated an organic content corresponding to an estimated PMNO loading of ∼15 wt % on the GNP surface (Figure 3c).

**Figure 3:**
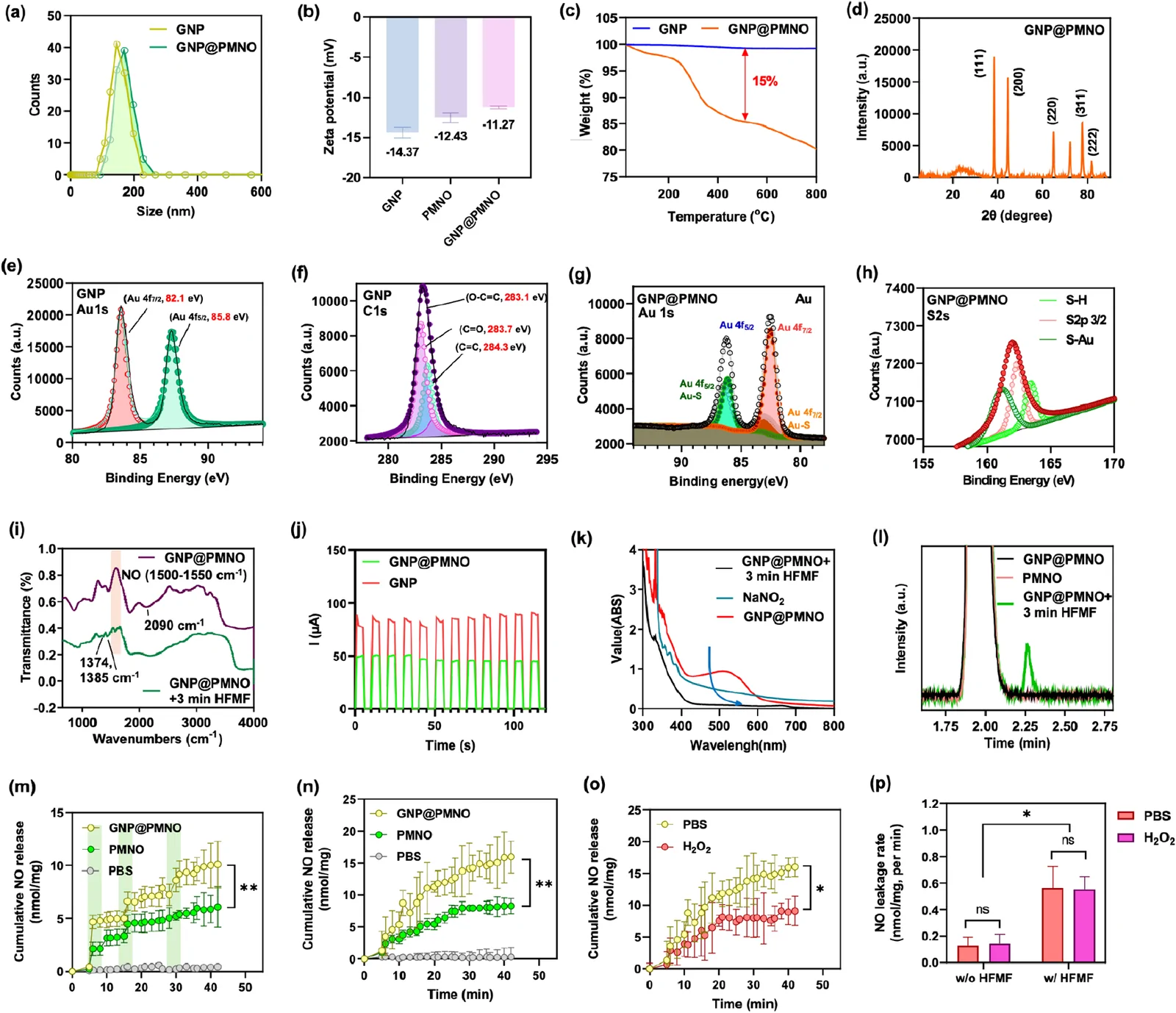
Electrical response and HFMF-triggered NO release from GNP@PMNO. (a) Size distribution of GNP and GNP@PMNO. (b) ζ-potential measurements of GNP, PMNO, and GNP@PMNO. (c) TGA of GNP and GNP@PMNO. (d) XRD patterns of GNP@PMNO. (e–h) XPS spectra of GNP and GNP@PMNO. (i) FTIR spectra of GNP@PMNO before and after HFMF treatment. (j) I–T responses of GNP and GNP@PMNO under intermittent HFMF stimulation. (k) NO release from GNP@PMNO under HFMF stimulation, quantified by the Griess assay using a microplate reader at 540 nm. (l) GC analysis of released NO from GNP@PMNO with HFMF treatment. (m) Cumulative NO release profile of GNP@PMNO under intermittent HFMF application (2.8 kW, 1 MHz, 3 min per cycle). The HFMF was alternately switched on (green shaded regions) and off (white regions). Data are presented as mean ± SD (*n* = 5). (n) Cumulative NO release profile of GNP@PMNO determined by DAF-FM fluorescence under intermittent HFMF stimulation (2.8 kW, 1 MHz), demonstrating stimulus-responsive NO generation (*n* = 5). (o) Cumulative NO release profile of GNP@PMNO under PBS and H_2_O_2_-containing solution under HFMF-on/off stimulation (2.8 kW, 1 MHz). (*n* = 5; mean ± s.d.; *p < 0.05; one-way ANOVA with Tukey’s multiple-comparison test). (p) Calculated NO leakage rate of GNP@PMNO under PBS and H_2_O_2_ condition. (*n* = 5; mean ± s.d.; *p < 0.05; one-way ANOVA with Tukey’s multiple-comparison test).

XRD analysis was subsequently employed to investigate the crystalline structure of GNP@PMNO (Figure 3d). The characteristic diffraction peaks correspond to the Au (111) and Au (200) planes, confirming a face-centered cubic (fcc) crystal structure (Figure S4). In addition, a broad peak at 23.5°—typically associated with an amorphous phase—can be attributed to the PM component. The surface elemental composition and chemical bonding states of GNP@PMNO were further investigated by high-resolution XPS (Figure 3e–h). The Au 4f spectrum displayed two characteristic peaks at binding energies of 82.1 and 85.8 eV, corresponding to the Au 4f_7/2_ and Au 4f_5/2_ orbitals of metallic gold (Au^0^), respectively. Compared with bare GNPs, a slight positive shift in the Au 4f binding energies was observed following PMNO conjugation, suggesting electronic interactions between the gold surface and sulfur-containing functional groups. Notably, the S 2p spectrum exhibited a distinct peak centered at 162.9 eV, which is characteristic of Au–S covalent bonding formed through thiol–gold interactions (Figure 3h). Collectively, these results provide compelling evidence for the successful conjugation of PMNO onto GNPs via stable Au–S bonds, while preserving the metallic gold core and crystalline structure.

To further evaluate the colloidal stability of GNP@PMNO, a particle stability assay was conducted. The results showed no obvious precipitation or aggregation during storage in PBS solution, as confirmed by SEM imaging and DLS measurements (Figure S5a–b). GNP@PMNO maintained a relatively stable morphology and hydrodynamic size distribution throughout the experimental conditions, indicating favorable dispersion stability. These findings suggest that GNP@PMNO is stabilized by steric effects arising from the platelet membrane (PM) coating rather than by electrostatic repulsion alone. The biomimetic membrane coating provides a hydrated and physically protective outer layer that helps minimize nanoparticle aggregation even under physiological ionic strength conditions.^63^ Furthermore, the well-preserved and reproducible current–time (I–T) responses under repeated HFMF stimulation demonstrated that although the electrical response gradually decreased over time (Figure S5c), the nanoparticles still retained their electrical responsiveness and functional stability after more than 2 weeks, indicating no obvious aggregation-induced signal deterioration. Collectively, these results support the favorable colloidal and functional stability of the GNP@PMNO system despite its relatively moderate ζ potential value.

### 2.5 Electrical Current Generation and NO Release from GNP@PMNO

The nitric oxide (NO) donor was generated through S-nitrosation of cysteine thiol (−SH) groups present on platelet membrane proteins, yielding S-nitrosothiol (−SNO) moieties that were immobilized on GNP@PMNO via Au–S anchoring. This configuration enables subsequent cleavage of the S–NO bond for NO release. To investigate whether HFMF stimulation induces S–NO bond dissociation, Fourier-transform infrared (FTIR) spectroscopy was performed on GNP@PMNO before and after HFMF treatment, while the I–T curves further demonstrated the electromagnetic responsiveness of GNP@PMNO under HFMF stimulation (Figure 3i). As shown in Figure 3i, characteristic vibrational bands associated with S–NO bond cleavage emerged following HFMF exposure, including peaks at 764 cm^–1^ (−S–N=) and 1505 cm^–1^ (−N=O), indicating disruption of the S–NO linkage. Additional spectral changes, including the appearance of a band at 2090 cm^–1^ attributed to C–N stretching in protein-derived moieties, further support the occurrence of chemical rearrangement accompanying NO release. Figure 3j further demonstrates that rapid current generation occurred synchronously with HFMF on/off switching even after PM coating, indicating that the PM layer did not significantly delay the HFMF responsiveness of the GNP core.

Importantly, these findings suggest that HFMF itself does not directly induce substantial NO release, but rather acts as an external trigger that activates the conductive GNP core to generate localized electromagnetic/electrical responses, which subsequently facilitate S–NO bond dissociation and transient NO liberation. In general, electric fields do not directly cleave chemical bonds; however, previous studies have suggested that localized electrical fields can indirectly influence weak chemical bonds by decreasing their dissociation energy and stabilizing the reaction products relative to the reactants because of differences in dipole moments.^64^ In systems containing labile S–NO bonds, localized electromagnetic/electrical interactions may alter the electron density distribution around the bond and thereby facilitate bond dissociation.^65,66^ Therefore, we propose that the conductive GNP core functions as an HFMF-responsive electrical transducer that converts external magnetic stimulation into localized interfacial electrical cues, thereby promoting controllable NO release from the PMNO shell.

To experimentally validate this mechanism, NO release from GNP@PMNO under HFMF stimulation was quantitatively evaluated using the Griess assay and gas chromatography (GC) (Figure S6). During the HFMF-on state, a pronounced absorbance peak at 540 nm was observed, indicating active NO generation from GNP@PMNO (Figure 3k). Consistently, GC analysis independently confirmed NO production upon HFMF application (Figure 3l). Under repeated HFMF exposure (2.8 kW, 1 MHz), burst-like NO release behavior was observed and was temporally synchronized with the on/off switching of the electromagnetic field (Figure 3m). Quantitative analysis revealed that 4.94 nmol mg^–1^ of NO was released during the initial HFMF-on period, whereas only minimal NO release (0.22 nmol mg^–1^) was detected during the HFMF-off state. Continuous HFMF on/off stimulation for 40 min resulted in a cumulative NO release of 10.55 nmol mg^–1^.

In contrast, the PMNO-only group exhibited substantially weaker and less responsive NO release behavior under identical HFMF on/off conditions because of the absence of conductive GNPs capable of responding to HFMF stimulation. These findings indicate that the observed NO release behavior is not solely governed by the intrinsic chemical properties of the NO donor itself, but is critically dependent on the GNP-mediated HFMF-responsive electrical transduction process. Specifically, the conductive GNP core likely converts electromagnetic stimulation into localized interfacial electrical cues, thereby facilitating rapid and controllable NO liberation from the PMNO shell. To further validate the controllable NO release behavior, fluorescence-based NO detection was performed using the NO-sensitive probe 4-amino-5-methylamino-2′,7′-difluorofluorescein (DAF-FM). As shown in Figure 3n, the cumulative NO release profile obtained from DAF-FM fluorescence closely matched the release trend measured by the Griess assay under intermittent HFMF stimulation, further confirming the gradual, controllable, and HFMF-responsive NO release capability of the GNP@PMNO system.

In addition, to assess potential off-target NO leakage under physiological conditions in the absence of magnetic stimulation, the NO release profile of GNP@PMNO was evaluated without HFMF exposure. The results showed negligible NO release from the GNP@PMNO group and no detectable NO release in the PBS control group under physiological conditions (Figure S7a). Furthermore, the NO release performance of GNP@PMNO under oxidative stress conditions was investigated in H_2_O_2_-containing solution with repeated HFMF on/off cycles (Figure 3o). NO release could still be effectively triggered by HFMF stimulation in the oxidative microenvironment, although the release efficiency was slightly lower than that observed under physiological conditions (PBS). As shown in Figure 3p, quantitative analysis of the NO leakage rate revealed minimal NO release in the absence of HFMF under both physiological and oxidative conditions, whereas HFMF stimulation significantly enhanced NO generation. Collectively, these results demonstrate that the GNP@PMNO platform maintains HFMF-responsive and controllable NO release behavior even in oxidative stress environments, while exhibiting minimal off-target leakage without external stimulation. These characteristics highlight its potential as a remotely activated gas-delivery platform for therapeutic applications requiring precise spatiotemporal control.

To further investigate the influence of HFMF parameters on NO release behavior, different HFMF intensities and frequencies were evaluated (Figure S7b–c). The results demonstrated that both HFMF intensity and frequency positively influenced NO release efficiency and release kinetics, with higher magnetic field strengths and frequencies leading to enhanced NO release responsiveness. This indicates that NO generation behavior can be precisely modulated through external HFMF parameters in a controllable manner. Notably, the selected experimental condition (2.8 kW, 1 MHz) exhibited the highest NO release efficiency and was therefore employed for subsequent biological evaluations. Collectively, these additional data further substantiate the tunable and HFMF-responsive NO release capability of the GNP@PMNO platform, supporting its potential as a controllable NO delivery system for synergistic therapeutic applications.

### 2.6 Cell Uptake and Intracellular Trafficking of GNP@PMNO

To investigate the effect of GNP@PMNO on cell activity, the number of surviving cells on NIH-3T3 cells (a murine fibroblast cell line; embryonic fibroblasts) was determined by using the MTT Presto Blue assay. In Figure 4a, both PM and PMNO exhibit no significant toxicity to the cells; in fact, PMNO can even promote cell proliferation at certain concentrations. GNP and GNP@PMNO also show low toxicity to the cells. When the cells were exposed to HFMF for 3 min, no significant toxicity was detected, suggesting that the NO and current did not have a significant impact on the cells (Figure S8). This indicates that GNP@PMNO does not cause harm to the cells and has good biocompatibility. In addition, the platelet membrane coating plays a crucial role in enhancing cellular uptake. It protects the nanoparticles from premature oxidation and facilitates their interaction with cells. To investigate this effect, a series of cellular uptake experiments were conducted using various concentrations of Cy5.5-labeled GNP and Cy5.5-labeled GNP@PMNO (Cy5.5 was loaded onto GNPs prior to platelet membrane modification). Both GNP and GNP@PMNO exhibited concentration-dependent cellular uptake, with increasing internalization observed as the concentration increased (Figures 4b and S9). Notably, GNP@PMNO demonstrated a further enhanced uptake efficiency compared to unmodified GNP, indicating the synergistic effect of platelet membrane and NO modification in promoting nanoparticle–cell interaction. However, the uptake appeared to reach a plateau at concentrations around 200 μg/mL, suggesting a potential saturation point. These findings highlight the improved cellular interaction of the modified nanoparticles.

**Figure 4:**
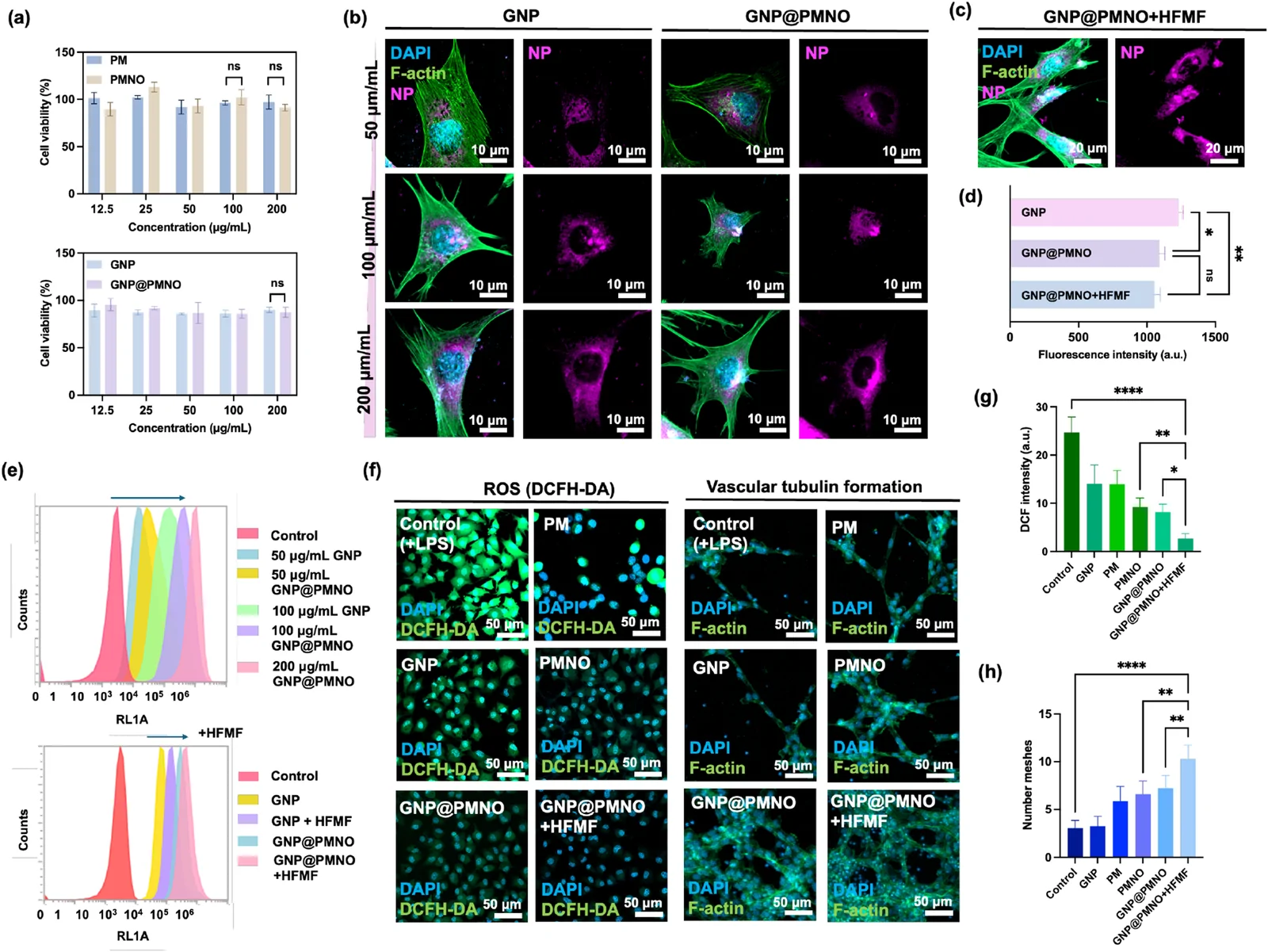
*In vitro* biocompatibility, cellular uptake, and intracellular ROS regulation. (a) Cell viability of NIH-3T3 cells after incubation with PM, PMNO, GNP, and GNP@PMNO, respectively (*n* = 5; mean ± s.d.; one-way ANOVA with Tukey’s multiple-comparison test). (b–c) Confocal laser scanning microscopy (CLSM) images showing cellular uptake of GNP, GNP@PMNO, and GNP@PMNO + HFMF (nuclei, blue; cytoskeleton, green; nanoparticles, purple). (d) Quantitative analysis of nanoparticle cellular uptake (*n* = 5; mean ± s.d.; *p < 0.05, **p < 0.01; one-way ANOVA with Tukey’s multiple-comparison test). (e) Flow cytometry analysis of cellular uptake under different treatments at varying concentrations. (f) Representative fluorescence images of intracellular ROS levels indicated by DCF fluorescence (green, DCFH-DA) and endothelial cytoskeleton staining (green, F-actin) for vascular network assessment. (g) Quantification of intracellular ROS levels based on DCF fluorescence intensity (*n* = 5; mean ± s.d.; *p < 0.05, **p < 0.01, ****p < 0.0001; one-way ANOVA with Tukey’s multiple-comparison test). (h) Quantification of mesh numbers to evaluate vascular network recovery in each group. (*n* = 5; mean ± s.d.; **p < 0.01, ****p < 0.0001; one-way ANOVA with Tukey’s multiple-comparison test).

For HFMF-treated groups (2.8 kW, 1 MHz, 3 min), the stimulation parameters were previously validated as non-cytotoxic and did not compromise cell viability. Following HFMF exposure, cells treated with GNP@PMNO exhibited markedly enhanced cellular uptake compared with their non-magnetic counterparts, as evidenced by fluorescence microscopy (Figures 4c and S10). Quantitative analysis of Cy5.5 fluorescence intensity further demonstrated that the GNP@PMNO + HFMF group displayed significantly higher intracellular fluorescence than cells treated with bare GNPs or GNP@PMNO alone, indicating enhanced nanoparticle internalization (Figure 4d). These observations were further corroborated by flow cytometry analysis. As shown in Figure 4e, nanoparticle uptake increased in a concentration-dependent manner across all treatment groups.

Importantly, confocal colocalization analysis further indicated that GNP@PMNO undergoes partial lysosomal escape upon HFMF stimulation, enabling redistribution of nanoparticles from endo/lysosomal compartments into the cytoplasm (Figure S11). This observation suggests that HFMF-triggered interfacial electromagnetic/electrical effects not only enhance cellular internalization but also facilitate intracellular trafficking beyond lysosomal sequestration. Such lysosomal escape behavior is critical, as it prevents excessive entrapment and degradation within acidic vesicles and increases the availability of GNP@PMNO in the cytosolic space. Consequently, this improved cytoplasmic accessibility provides a more favorable intracellular environment for HFMF-responsive NO release, thereby maximizing its interaction with intracellular ROS and contributing to redox regulation.^67^

To further validate the HFMF-triggered intracellular NO release capability of the GNP@PMNO system, intracellular NO production was assessed using the DAF-FM DA fluorescent probe. DAF-FM DA is a cell-permeable probe that enables detection of intracellular NO via its reaction with NO-derived nitrosating species (e.g., N_2_O_3_), which are generated from NO autoxidation.^68^ As shown in Figure S12, cells treated with GNP@PMNO under HFMF stimulation exhibited markedly enhanced green fluorescence compared with control groups, indicating elevated intracellular NO levels. In contrast, negligible fluorescence was observed in the absence of HFMF, confirming that NO release is tightly regulated by magnetic field activation. Importantly, neither the GNP + HFMF group nor the PMNO + HFMF group alone generated comparable fluorescence signals, indicating that the combination of the conductive GNP core and the PMNO shell is essential for efficient HFMF-triggered NO release. These results demonstrate that the GNP@PMNO platform enables controllable, HFMF-responsive NO delivery at the cellular level, consistent with its proposed electrically transduced release mechanism.

### 2.7 *In Vitro* Oxidative Stress-Attenuating and Vascular Network Formation

To confirm the redox-modulating effects associated with the HFMF-triggered intracellular NO release capability of the GNP@PMNO system, both chemical assays and cellular studies were performed. Initially, redox-regulatory activity was assessed using TMB and ABTS radical assays. In the TMB assay, where oxidation generates a blue chromophore with characteristic absorbance at 652 nm, the GNP@PMNO + HFMF group exhibited substantially lower absorbance compared with the control group, indicating effective suppression of oxidative reactions (Figure S13a). Similarly, in the ABTS assay, the characteristic absorbance of the oxidized ABTS·^+^ radical at 734 nm was markedly reduced in the GNP@PMNO + HFMF group, further corroborating its enhanced redox-regulatory activity (Figure S13b).

The results demonstrate that the combination of the conductive GNP core and PMNO shell enables effective HFMF-responsive redox modulation through controllable NO release. Specifically, the localized electromagnetic/electrical stimulation generated by the GNP core under HFMF irradiation facilitates intracellular NO liberation, which subsequently contributes to reactive oxygen species modulation and attenuation of oxidative stress.^69^ Collectively, these findings support the potential of the GNP@PMNO platform to regulate intracellular redox homeostasis under oxidative stress conditions.

To further evaluate the regulatory effect of GNP@PMNO on intracellular oxidative stress, intracellular ROS levels were assessed following nanoparticle treatment. DCFH-DA is a commonly used intracellular redox probe that is oxidized by ROS to generate the green fluorescent product dichlorofluorescein (DCF), enabling visualization of intracellular oxidative stress.^70^ Cells treated with GNP@PMNO exhibited a pronounced reduction in intracellular ROS compared with untreated controls and cells treated with bare GNPs, indicating that PMNO functionalization confers effective antioxidative capability (Figure 4f,g). This ROS-scavenging effect is primarily attributed to the transient release of nitric oxide from S-nitrosylated platelet membrane proteins, which can directly neutralize reactive oxygen species and suppress oxidative stress–associated signaling pathways. Notably, under HFMF stimulation, the ROS-regulatory effect of GNP@PMNO was further enhanced (Figure 4f). The magnetically induced electrical or polarization effects of the GNP core may facilitate NO release kinetics and improve intracellular distribution, thereby reinforcing redox homeostasis. In addition, the platelet membrane coating enhances cellular internalization and biocompatibility, enabling more efficient intracellular delivery of NO and prolonged antioxidative action.

An *in vitro* tube formation assay was performed to investigate the recovery of vascular network integrity in endothelial cells under oxidative stress following GNP@PMNO treatment. 3B11 murine endothelial cells were employed due to their robust capacity to form capillary-like structures within extracellular matrix environments. In Figure 4f (right), cells treated with GNP@PMNO exhibited markedly enhanced tube formation, characterized by increased branch length, junction density, and overall architectural integrity, compared with untreated and bare GNP-treated groups. This pro-angiogenic effect is primarily attributed to the NO released from S-nitrosylated platelet membrane proteins on GNP@PMNO, which is known to promote endothelial cell survival, migration, and angiogenic signaling while concurrently mitigating oxidative stress–induced endothelial dysfunction.^71^ By suppressing intracellular ROS accumulation, GNP@PMNO preserves cytoskeletal organization and intercellular junction stability, thereby facilitating the re-establishment of capillary-like networks under oxidative conditions. Notably, the GNP@PMNO + HFMF group demonstrated the highest number of meshes and the most interconnected vascular network among all groups (Figure 4h). This further enhancement is likely driven by HFMF-triggered NO release and magnetoelectric-assisted cellular interactions, which synergistically amplify endothelial activation and angiogenic responses. Collectively, these findings indicate that GNP@PMNO, particularly under HFMF stimulation, effectively restores vascular network integrity under oxidative stress, underscoring its potential to promote angiogenesis and microvascular repair in oxidative injury–associated pathological conditions.

### 2.8 *In Vitro* Neural Differentiation Study

Next, to evaluate the neural repair potential of the developed materials, neural stem cell (NSC) differentiation was investigated. In this context, GNP@PMNO was designed not only as a delivery platform but also as an active microenvironmental modulator to regulate NSC fate (Figure 5a). NO is a well-recognized neuromodulatory molecule that promotes NSC differentiation and neurite outgrowth by regulating intracellular redox balance and activating downstream neurogenic signaling pathways.^72^ In parallel, HFMF-induced magnetoelectric or surface polarization effects of the GNP core may further contribute to membrane potential modulation and intracellular signaling activation, thereby synergistically enhancing differentiation efficiency. Primary NSCs extracted from embryos are used for further evaluation of the therapeutic stimulation’s function. The steps for extracting neural stem cells are shown in Figure 5b. Briefly, NSCs were seeded in 24 wells for 3 days, and then materials from different groups were added, co-cultured with the materials for 7 days. The NSC differentiation experiment was divided into five groups: (1) Control group, (2) GNP group, (3) GNP+HFMF group, (4) GNP@PMNO group, (5) GNP@PMNO+HFMF group. HFMF at a power of 2.8 kW and a frequency of 1 MHz was applied for 3 min daily until the NSCs were fixed.

**Figure 5:**
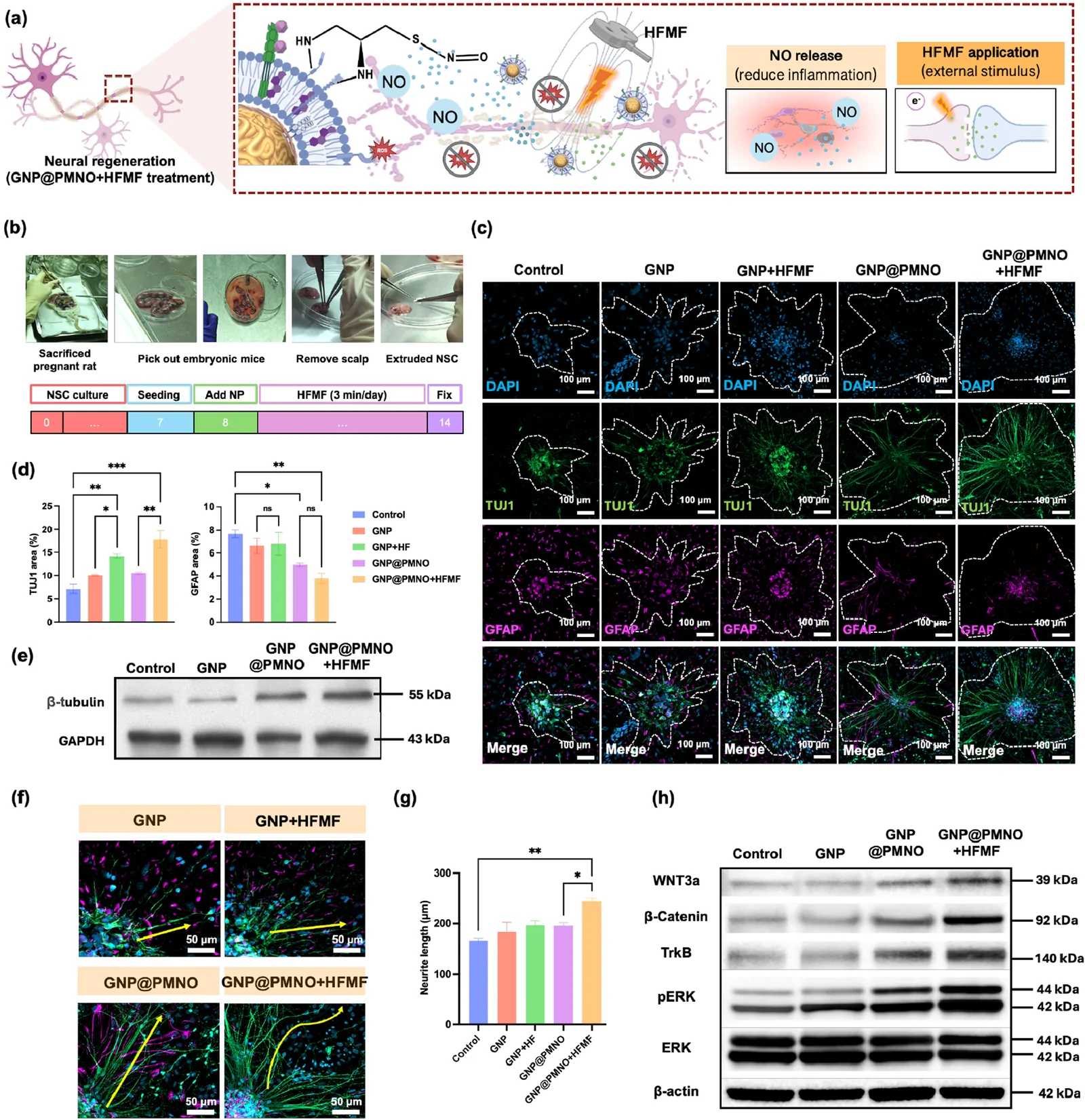
*In vitro* evaluation of HFMF-stimulated neural stem cell (NSC) differentiation. (a) Schematic illustration of GNP@PMNO combined with HFMF stimulation applied to primary neural cells to promote neurite outgrowth. (b) Experimental workflow illustrating primary NSC isolation from embryonic cerebral cortex and the differentiation timeline. (c) Confocal fluorescence images of NSCs differentiated from embryonic cortical neurospheres after 7 days in culture, showing immunostaining for TUJ-1 (green, neurons) and GFAP (purple, astrocytes). (d) Quantitative analysis of TUJ-1– and GFAP-positive cells migrated from neurospheres into the surrounding region (*n* = 5; mean ± s.d.; *p < 0.05, **p < 0.01, ***p < 0.001; one-way ANOVA with Tukey’s multiple-comparison test). (e) Western blot analysis of neuronal βIII-tubulin (TUJ-1) expression in NSCs under different treatment conditions. (f) High-magnification images showing neuronal differentiation morphology of NSCs treated with different groups. (g) Quantification of neurite length to compare neurite outgrowth among different treatment groups (*n* = 5; mean ± s.d.; *p < 0.05, **p < 0.01; one-way ANOVA with Tukey’s multiple-comparison test). (h) Western blot analysis of key proteins associated with the MAPK and Wnt signaling pathways in NSCs under different treatment conditions.

In the control group, strong glial fibrillary acidic protein (GFAP) immunoreactivity, indicative of astrocytic differentiation, and weak neuron-specific class III β-tubulin (TUJ-1) expression were observed, reflecting the intrinsic tendency of NSCs to preferentially differentiate into astrocytes under unstimulated conditions (Figure 5c,d). In contrast, GNP@PMNO treatment markedly biased neural stem cell (NSC) fate toward neuronal differentiation, as evidenced by a significant increase in TUJ-1 expression accompanied by a pronounced reduction in GFAP signal intensity (Figure 5c–d). Notably, the GNP@PMNO + HFMF group exhibited the highest TUJ-1 expression, indicating the most robust neuronal differentiation among all treatment groups (Figure 5e). Collectively, these results indicate that HFMF-activated GNP@PMNO synergistically integrates NO-mediated biochemical signaling with magnetoelectrically induced bioelectronic cues, thereby promoting neuronal lineage commitment while suppressing excessive astrocytic differentiation.

Furthermore, to ensure that the magnetic field itself did not influence cellular behavior, an additional “HFMF only” control group was included in the *in vitro* neural differentiation assays (Figure S14a). The results demonstrated that HFMF treatment alone did not significantly enhance neurite outgrowth or increase the percentage of TUJ-1-positive neuronal cells compared with the control group. These findings indicate that the applied HFMF conditions do not substantially affect neural differentiation behavior in the absence of nanoparticles. In contrast, significant neuronal differentiation and neurite extension were primarily observed in the nanoparticle-treated groups under HFMF stimulation, suggesting that the therapeutic effects originate from the HFMF-responsive bioelectronic activity of the GNP-based platform rather than from magnetic field exposure alone.

High-magnification fluorescence imaging further revealed that neuronal differentiation predominantly occurred within the central regions of the neurospheres (Figures 5f and S14b). Quantitative analysis demonstrated that the GNP@PMNO-treated group exhibited a substantially higher density of TUJ-1–positive neurons and more extensive neurite outgrowth compared with the control group (Figure 5g), indicating enhanced neuronal maturation. Notably, the application of HFMF stimulation further amplified these effects, likely through the activation of multiple electrically responsive signaling pathways.^73^ Specifically, HFMF-induced bioelectronic stimulation is proposed to promote calcium influx through voltage-gated calcium channels, thereby activating calcium-dependent signaling pathways involved in neuronal maturation and neurite extension. Concurrently, the generated electromagnetic cues may activate the MAPK signaling cascade, including ERK and p38 pathways, which regulate neuronal differentiation and cytoskeletal remodeling (Figure 5h).^74,75^ In addition, modulation of Wnt signaling further promotes the differentiation of neural stem cells toward neuronal lineages (Figure 5h).

Consistent with these signaling events, the GNP@PMNO + HFMF group exhibited increased expression of neurotrophic factors, including brain-derived neurotrophic factor (BDNF), β–nerve growth factor (β-NGF), ciliary neurotrophic factor (CNTF), and glial cell line–derived neurotrophic factor (GDNF), as well as the angiogenesis-related factors vascular endothelial growth factor A (VEGF-A) and transforming growth factor β (TGF-β) (Figure S15). These changes suggest the establishment of a pro-regenerative microenvironment that supports neuronal survival, neurite development, and neurovascular remodeling. Collectively, these findings indicate that HFMF-responsive bioelectronic stimulation, together with NO-mediated microenvironmental modulation, promotes neural differentiation and neurite outgrowth through the coordinated activation of multiple regenerative signaling pathways.

### 2.9 *In Vivo* Study of GNP@PMNO on TBI

Having demonstrated effective cell infiltration and neuronal differentiation *in vitro*, GNP@PMNO was subsequently evaluated in an *in vivo* TBI mouse model. TBI was induced in 7-week-old female C57BL/6 mice by drilling a partial hole in the left cerebral hemisphere to a depth of 1.5 mm. One hour post-injury, GNP@PMNO was administered via intravenous injection. Mice in the electromagnetic stimulation groups received daily treatment with a HFMF at a power of 2.8 kW and frequency of 1 MHz for 3 min per session until sacrifice (Figure 6a). The objective of this experiment was to develop a nanoparticle system capable of targeting brain injury sites through systemic circulation, releasing NO, and generating electrical signals to modulate the local neural microenvironment, thereby facilitating tissue repair and regeneration (Figure 6b). To assess the therapeutic effects, immunohistochemical analyses were performed to investigate cellular responses in the peri-lesion area at 7- and 30-days post-injury, with a particular focus on neuroinflammation and cellular remodeling (Figure 6c,d). Specific markers were used to assess astrocyte proliferation, microglial activation, and overall neurovascular response. Mice were randomly assigned to five experimental groups: (1) Control, (2) GNP alone, (3) GNP + HFMF, (4) GNP@PMNO, and (5) GNP@PMNO + HFMF. For the HFMF-treated groups, real-time thermal monitoring was performed during magnetic stimulation (Figure S16a–b). The temperature remained below 34 °C after 3 min of continuous HFMF irradiation, indicating minimal bulk heat generation during treatment. This result suggests that the applied electromagnetic energy was primarily converted into localized electrical stimulation rather than macroscopic thermal dissipation, thereby supporting the thermal safety of the HFMF treatment conditions.

**Figure 6:**
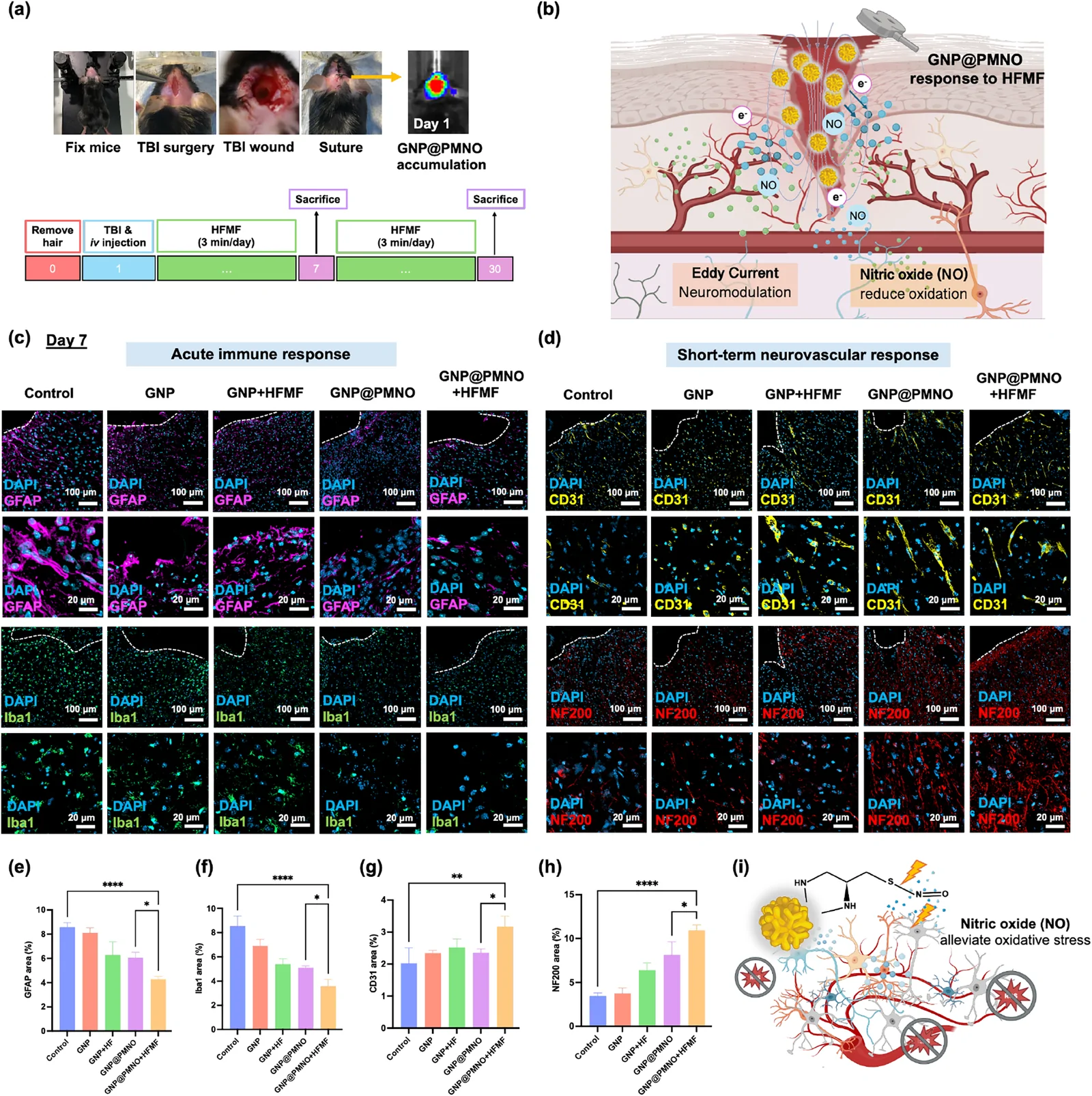
*In vivo* therapeutic evaluation of GNP@PMNO in a TBI model. (a) Top: Schematic illustration of the TBI surgical procedure and treatment protocol. Bottom: Experimental timeline showing nanoparticle administration, HFMF stimulation, and subsequent tissue harvesting for histological analysis. (b) Illustration of the therapeutic strategy employing GNP@PMNO under HFMF stimulation to enable targeted NO delivery in TBI. (c–d) CLSM images of peri-lesional brain tissue at 7 days post-injury, representing short-term pathological responses. Astrocytes (purple, GFAP), microglia/macrophages (green, Iba1), blood vessels (yellow, CD31), and neurofilaments (red, NF200) were immunostained to evaluate reactive gliosis, neuroinflammation, angiogenesis, and axonal integrity, respectively. Nuclei were counterstained with DAPI (blue). (e–h) Quantitative analysis of GFAP-positive scar area, Iba1-positive inflammatory area, NF200-positive neuronal area, and CD31-positive vascular area at the lesion site (*n* = 5; mean ± s.d.; *p < 0.05, **p < 0.01, ****p < 0.0001; one-way ANOVA with Tukey’s multiple-comparison test). (i) Schematic illustration depicting the proposed mechanism whereby HFMF-activated GNP@PMNO enables localized NO release to suppress neuroinflammation, mitigate glial scar formation, and improve the injury microenvironment following TBI.

Glial fibrillary acidic protein (GFAP) staining was used to evaluate astrocyte activation and glial scar formation in the peri-injury region. Excessive proliferation of reactive astrocytes can lead to glial scar formation, which serves as a physical and biochemical barrier that inhibits the infiltration of neural progenitor cells and impairs tissue regeneration. In addition, the absence of new cell infiltration, combined with apoptosis of existing cells, can result in cavity expansion and further neurological dysfunction. In Figure 6c, a dense accumulation of GFAP-positive astrocytes was observed around the lesion site in the untreated control group, indicating prominent glial scarring. In contrast, the GNP@PMNO+HFMF group exhibited a visibly reduced astrocytic response. Figure 6e confirmed a significant decrease in astrocyte density in this group, suggesting that the combined effects of GNP-mediated electrical stimulation and NO release may attenuate reactive gliosis, thereby reducing scar formation and facilitating brain tissue repair following traumatic injury. The neuroinflammatory response following TBI was evaluated by immunostaining for ionized calcium-binding adaptor molecule 1 (Iba1), a marker for activated microglia and infiltrating macrophages. As key mediators of the innate immune response, excessive activation of these cells can exacerbate secondary injury and impede tissue regeneration. Figure 6c presents confocal images of Iba1 expression, highlighting the accumulation and activation of microglia/macrophages in the peri-lesion area. Quantitative analysis (Figure 6f) demonstrated a progressive reduction in the Iba1-positive area across the treatment groups, indicating attenuation of microglial activation after TBI. Notably, the GNP@PMNO + HFMF group exhibited the most significant suppression of neuroinflammatory responses, showing an approximately 2.2-fold decrease in Iba1-positive microglial content compared with the control group. Statistical analysis confirmed a significant reduction in activated microglia following the combined treatment. These results suggest that the combination of electrical stimulation and NO release attenuates the inflammatory response, thereby contributing to a more favorable microenvironment for neural repair after TBI.

To evaluate angiogenesis in the peri-injury region, immunofluorescent staining for cluster of differentiation 31 (CD31), an endothelial cell marker, was performed to visualize and quantify neovascularization. TBI often leads to vascular disruption and endothelial cell apoptosis, contributing to impaired perfusion and limiting the regenerative capacity of the injured tissue. Therefore, the extent and quality of vascular regeneration can serve as an important indicator of tissue recovery. In Figure 6d, confocal microscopy revealed a disrupted vascular network in the control group, whereas groups treated with GNP or GNP@PMNO exhibited enhanced blood vessel density around the lesion site. Notably, the GNP@PMNO + HFMF group displayed a more continuous and organized vascular structure, suggesting improved angiogenic response under eddy current generation. The quantitative analysis of CD31 fluorescence intensity (Figure 6g) revealed a significant increase in vascular density in the GNP@PMNO+HFMF group compared to other groups. This enhancement is attributed to the combined effects of GNP-mediated electrical stimulation and HFMF-triggered NO release. Neurofilament 200 (NF200) was used as a marker to evaluate axonal regeneration in the peri-injury region.

In Figure 6d, the control group exhibited limited NF200 expression, indicating poor spontaneous axonal recovery. In contrast, the GNP@PMNO-treated groups showed markedly enhanced NF200 immunoreactivity, with the GNP@PMNO + HFMF group displaying the most pronounced increase. Quantitative analysis (Figure 6h) further revealed a significant 3.1-fold increase in the NF200-positive area in the GNP@PMNO + HFMF group compared with the control group at 7 days post-injury, indicating substantial enhancement of axonal structural recovery following treatment. This enhancement is likely attributable to the synergistic effects of HFMF-induced localized bioelectronic stimulation and NO-mediated neuroregulatory signaling.

To verify that tissue regeneration stems uniquely from the fully assembled BEA platform, we evaluated the single-control cohorts (HFMF-only, GNP-only, and PMNO-only) (Figure S16c). Despite their excellent biocompatibility, these individual components demonstrated clear functional limitations. The HFMF-only group lacked the nanoparticle transducers required to generate localized electrical cues, while standalone GNP-only and PMNO-only treatments failed to trigger robust neurogenesis or axonal remodeling due to the absence of active bioelectronic stimulation or controllable NO delivery. Ultimately, these single-component limitations underscore that the complex inhibitory lesion microenvironment cannot be overcome individually, proving that neural stem cell mobilization and subsequent axonal sprouting require the multi-modal synergy of the integrated GNP@PMNO platform under HFMF activation.

Furthermore, to substantiate the regenerative effects of the BEA platform, we additionally performed immunohistochemical staining for Nestin, a definitive marker for undifferentiated endogenous neural stem/progenitor cells,^76^ to assess the mobilization of host regenerative pools (Figure S17). The results demonstrated significantly elevated Nestin-positive signals in the GNP@PMNO-treated group compared with the GNP group, while HFMF stimulation further amplified Nestin expression levels, indicating a targeted awakening and physical recruitment of endogenous progenitor cells flanking the peri-lesion cortical border. Following this upstream progenitor mobilization, we subsequently performed immunohistochemical staining for growth-associated protein 43 (GAP-43), a well-established marker of axonal sprouting, neuronal regeneration, and neural plasticity following CNS injury.^77^ The results demonstrated significantly elevated GAP-43-positive signals in the GNP@PMNO-treated group compared with the GNP group, while HFMF stimulation further amplified GAP-43 expression (Figure S18).

Mechanistically, this sequential enhancement of progenitor activation and active sprouting likely stems from the combined interplay between HFMF-responsive bioelectronic stimulation and controllable NO release. Localized electrical cues from HFMF-activated GNPs promote endogenous progenitor proliferation, neuronal excitability, and neurite extension, while NO modulates the microenvironment to support axonal remodeling.^78^ Biologically, this transient NO release profile is an intentional, stage-matched therapeutic strategy rather than a system limitation. While acute NO delivery quenches ROS, prevents microvascular collapse, and suppresses early neuroinflammation, continuous exposure risks forming toxic peroxynitrite (ONOO−) and causing secondary nitrosative damage.^79^ To elucidate the molecular microenvironment driving this cascade, *in vivo* protein microarray analysis (Figure S19) confirmed that acute GNP@PMNO + HFMF treatment significantly upregulates long-acting neurotrophic (β-NGF) and angiogenic (TGF-β) mediators. This enriched paracrine profile demonstrates that acute NO release functions as an upstream catalytic trigger rather than a continuously required drug. Coupled with Nestin-mediated progenitor recruitment and GAP-43-tracked growth cone formation, these concurrent molecular and cellular responses confirm a coordinated regenerative cascade induced by the BEA platform.

Collectively, histological analyses of brain sections at 7 days post-injury demonstrate that HFMF-activated GNP@PMNO exerts a pronounced early-stage neuroprotective effect following TBI (Figure 6i). At this acute phase, treatment with GNP@PMNO under HFMF stimulation effectively attenuated reactive gliosis and microglial activation, as evidenced by the reduced GFAP- and Iba1-positive areas, indicating suppression of excessive neuroinflammatory responses that are known to drive secondary tissue damage. In parallel, the preservation and partial restoration of vascular and axonal architecture—reflected by increased CD31-positive neovascularization and enhanced NF200 expression—suggest that GNP@PMNO treatment helps maintain the structural integrity of both the cerebrovascular and neuronal networks surrounding the lesion site. These early regenerative trends are likely mediated by the synergistic actions of HFMF-induced electrical stimulation and localized nitric oxide release, which together modulate inflammatory signaling, improve endothelial function, and support neuronal survival. Importantly, these findings indicate that GNP@PMNO establishes a permissive microenvironment during the acute post-injury window, laying a critical foundation for subsequent vascular remodeling, axonal regeneration, and long-term functional recovery.

### 2.10 *In Vivo* GNP@PMNO Treatment 30 Days Post-Injury

Based on the proposed therapeutic mechanism illustrated in Figure 7a, HFMF-activated GNP@PMNO orchestrates a multistage neurorepair process following TBI. Upon intracerebral administration, GNP@PMNO enables on-demand NO release and localized electrical stimulation under HFMF exposure. In the acute phase, NO mitigates oxidative stress by scavenging excess ROS and suppresses neuroinflammation through modulation of the neuroimmune microenvironment.^80^ These early effects subsequently facilitate angiogenesis, neurogenesis, and neural network remodeling in the chronic phase, thereby supporting tissue repair and functional recovery, which together account for the long-term therapeutic benefits observed at 30 days post-injury (Figure 7b,c).

**Figure 7:**
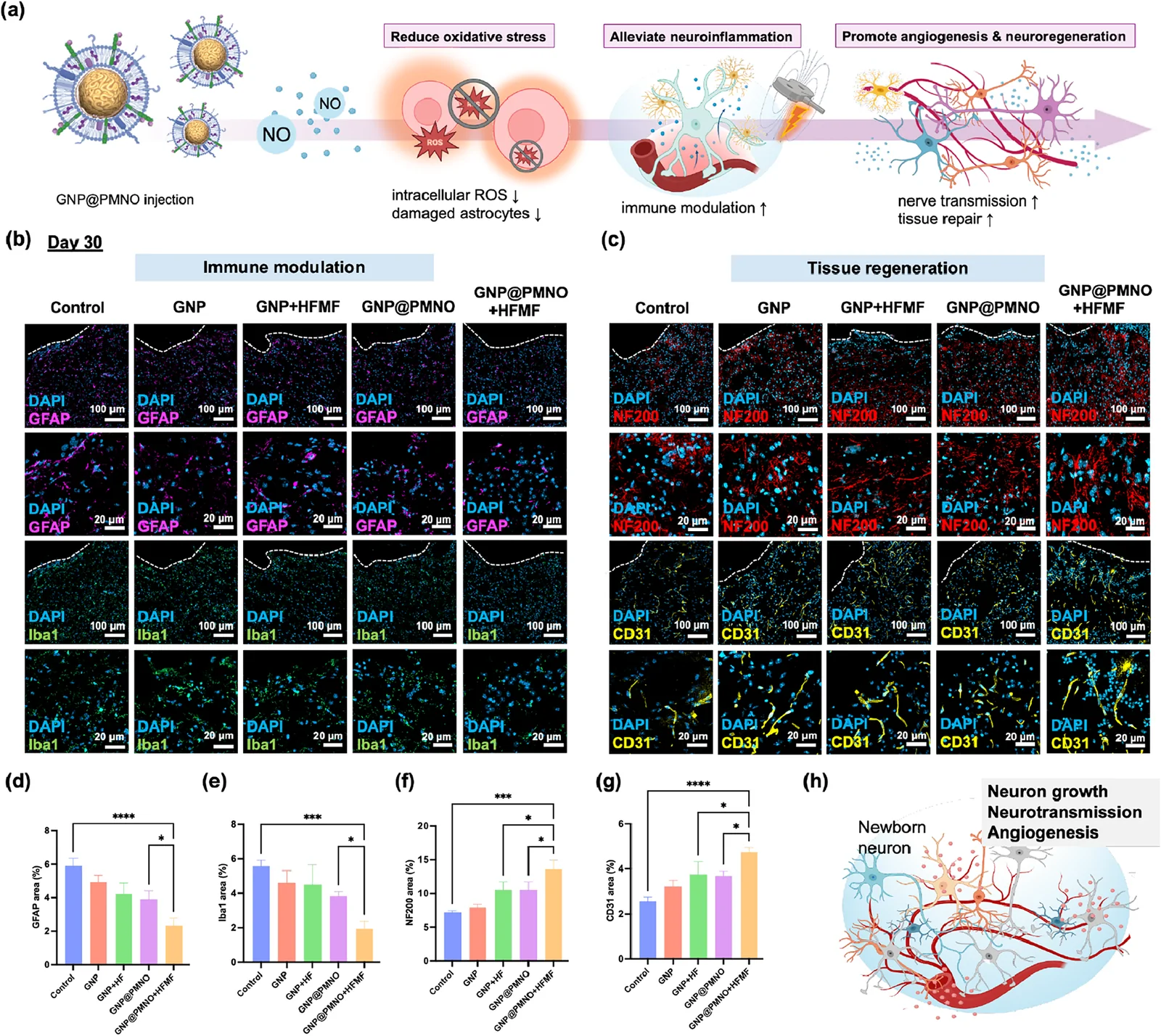
Long-term *in vivo* therapeutic effects of GNP@PMNO at 30 days post-injury. (a) Schematic illustration of the stepwise therapeutic mechanism of GNP@PMNO administration following TBI, highlighting the progression from early ROS attenuation and neuroinflammation suppression to subsequent tissue repair and remodeling. (b–c) CLSM images of peri-lesional brain tissue at 30 days post-injury, representing long-term pathological outcomes. Astrocytes (purple, GFAP), microglia/macrophages (green, Iba1), neurofilaments (red, NF200), and blood vessels (yellow, CD31) were immunostained to assess glial scarring, neuroinflammation, axonal regeneration, and angiogenesis, respectively. Cell nuclei were counterstained with DAPI (blue). (d–g) Quantitative analysis of GFAP-positive scar area, Iba1-positive inflammatory area, CD31-positive vascular area, and NF200-positive neuronal area at the lesion site (*n* = 5; mean ± s.d.; *p < 0.05, ***p < 0.001, ****p < 0.0001; one-way ANOVA with Tukey’s multiple-comparison test). (h) Schematic summary illustrating the proposed long-term tissue repair process induced by GNP@PMNO under HFMF stimulation, including sustained suppression of chronic inflammation, enhanced vascular reconstruction, and progressive neural network restoration.

In Figure 7b, by 30 days post-injury, inflammatory responses had largely subsided across all groups; however, distinct differences in chronic glial remodeling remained. The control group exhibited dense accumulation of GFAP-positive astrocytes and persistent glial scar formation at the lesion site, indicative of sustained reactive gliosis. In contrast, the GNP@PMNO-treated groups, particularly under HFMF stimulation, exhibited a markedly reduced and more dispersed distribution of GFAP-positive astrocytes, suggesting alleviated reactive astrogliosis. Quantitative analysis (Figure 7d) further demonstrated an approximately 2.3-fold reduction in GFAP-positive area in the GNP@PMNO + HFMF group compared with the control group, indicating a significant long-term suppression of astrocyte activation. Consistently, Iba1 staining demonstrated reduced microglial/macrophage presence in treated groups, with the GNP@PMNO + HFMF group showing an approximately 3% decrease in Iba1-positive area relative to the control (Figure 7e). Collectively, these results indicate that HFMF-activated GNP@PMNO exerts sustained immunomodulatory effects during the chronic phase of TBI, limiting glial scar maturation and neuroinflammation, thereby promoting a more permissive environment for long-term brain tissue repair.

At 30 days post-injury, TBI induced neuronal loss and vascular disruption remained evident in the control group, reflecting limited spontaneous recovery. Given the critical role of neurovascular reconstruction in long-term tissue repair, both axonal regeneration and vascular remodeling were evaluated at this chronic stage. In Figure 7c, the GNP@PMNO + HFMF group exhibited pronounced neurofilament regeneration in the peri-lesion region, suggesting sustained neuronal outgrowth and structural reorganization extending toward the injury core. Quantitative analysis further confirmed that HFMF stimulation significantly enhanced neuronal regeneration, with the GNP@PMNO + HFMF group showing the highest level of neural marker expression among all treatment groups (Figure 7f).

In parallel, angiogenic remodeling was markedly improved in the GNP@PMNO + HFMF group at 30 days post-injury. Confocal imaging revealed the presence of elongated and interconnected CD31-positive vessels exceeding 80 μm in length, whereas the control group displayed sparse and discontinuous vascular structures (Figure 7c). Quantitative analysis revealed an approximately 1.8-fold increase in CD31-positive area in the GNP@PMNO + HFMF group compared with the control group (Figure 7g), indicating significantly enhanced angiogenic activity following treatment. This vascular improvement is likely attributable to the synergistic effects of nitric oxide release and HFMF-induced bioelectrical stimulation, which together promote endothelial cell activation, vascular network formation, and maturation, ultimately supporting improved neurovascular coupling within the injured tissue. Together, these findings demonstrate that GNP@PMNO combined with HFMF facilitates coordinated neuronal and vascular regeneration during the chronic phase of TBI, providing structural support for long-term tissue repair and functional recovery (Figure 7h).

### 2.11 *In Vivo* Biodistribution, Clearance, and Biosafety of GNP@PMNOs

To evaluate the *in vivo* biodistribution, and clearance behavior of GNP and GNP@PMNO, near-infrared fluorescence imaging was performed using an IVIS system. Cy5.5-labeled nanoparticles were intravenously administered, and fluorescence signals were monitored over time. As shown in Figure S20, an initial broad signal distribution was observed, with pronounced liver accumulation at 4 h post-injection, consistent with reticuloendothelial system uptake. Fluorescence intensity gradually diminished across major organs, becoming nearly undetectable by day 7. In the brain, GNP@PMNO displayed significantly stronger fluorescence and prolonged retention up to day 14 compared to bare GNPs (Figures 8a,b and S21), confirming enhanced BBB crossing and lesion targeting mediated by the platelet membrane cloak.

**Figure 8:**
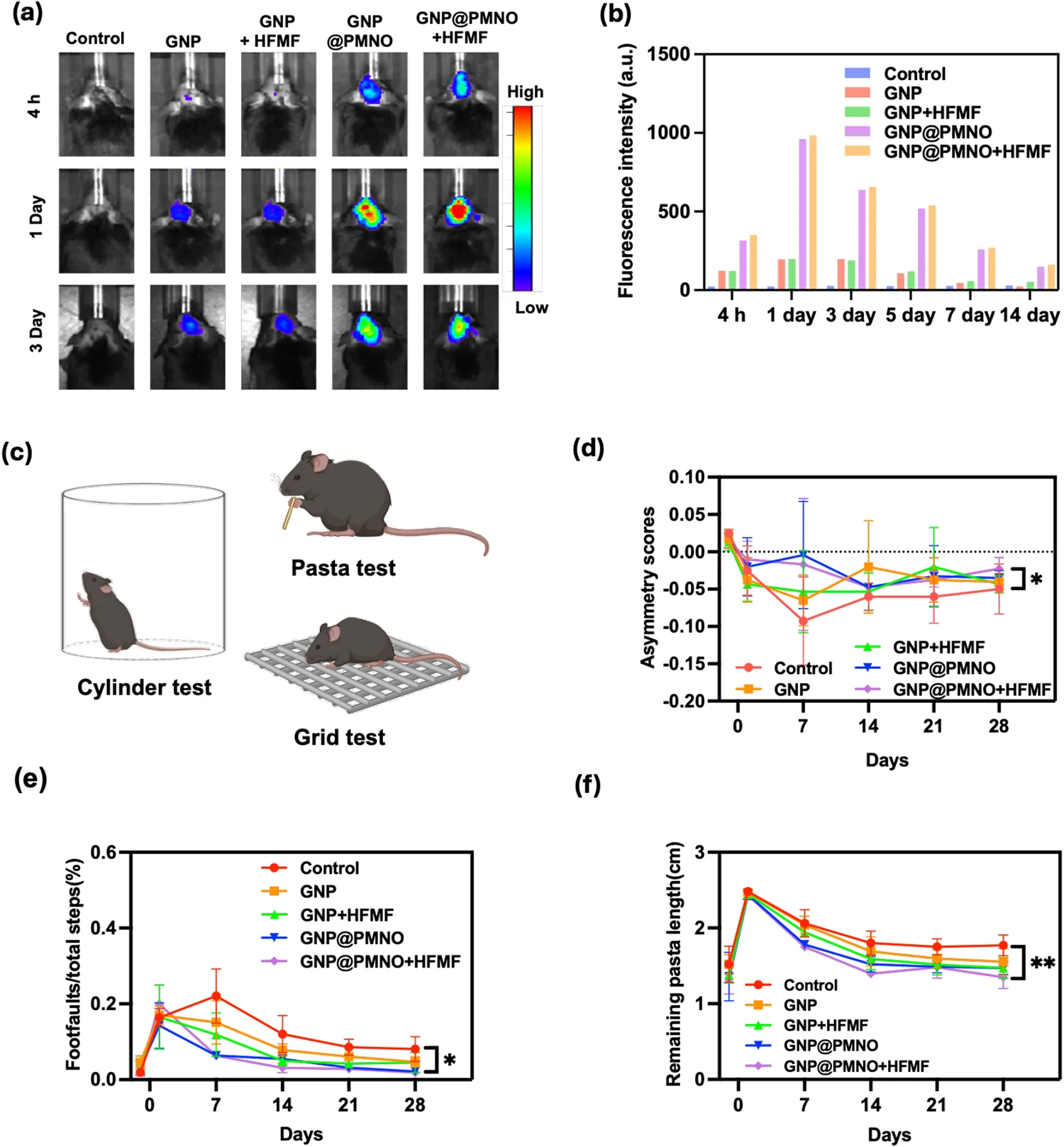
*In vivo* biodegradation and functional recovery following GNP@PMNO treatment. (a) *In vivo* imaging system (IVIS) tracking of nanoparticle distribution and degradation over time in Control, GNP, GNP+HFMF, GNP@PMNO, and GNP@PMNO+HFMF groups. (b) Quantitative analysis of fluorescence intensity across treatment groups, indicating nanoparticle retention and clearance. (c) Representative images from behavioral tests, including the cylinder, grid, and pasta handling tests, performed after treatment. (d-f) Quantitative evaluation of motor function recovery in the different groups based on behavioral assessments (*n* = 8; mean ± SD; *p < 0.05, **p < 0.01; one-way ANOVA with Tukey’s multiple comparison test).

To observe long-term systemic biosafety, both structural and functional parameters were assessed at 30 days post-treatment. H&E staining of the brain and major metabolic organs revealed no histopathological abnormalities, cellular toxicity, or tissue damage across all treatment groups (Figure S22). Concurrently, quantitative serum biochemical analysis demonstrated that key liver function indicators—alanine aminotransferase (ALT) and aspartate aminotransferase (AST)—remained well within normal physiological ranges without significant differences among groups (Figure S23), validating functional hepatic safety. Furthermore, HFMF exposure induced no detectable adverse tissue responses.

Although the GNP core remains a non-biodegradable scaffold, elemental gold (Au^0^) is inherently bioinert and exhibits minimal chronic toxicity.^81,82^ This bioinertness is supported by the gradual clearance of nanoparticles without bioaccumulation. Crucially, the absence of organ toxicity (Figure S22 and S23) and the ∼2.2-fold reduction in Iba1+ reactive microglial coverage in the GNP@PMNO + HFMF cohort (Figure 7e) demonstrate that residual gold cores do not induce foreign-body reactions, hepatotoxicity, or chronic neuroinflammation. Overall, the restoration of microglial homeostasis and uncompromised systemic health demonstrate the outstanding long-term biosafety of the BEA platform.

### 2.12 Animal Behavior Tests

To evaluate functional recovery following TBI, behavioral assessments were performed using seven-week-old female C57BL/6 mice. Animals were randomly assigned to five experimental groups: (1) control (untreated), (2) GNP, (3) GNP + HFMF, (4) GNP@PMNO, and (5) GNP@PMNO + HFMF (*n* = 8 per group). All behavioral experiments and data analyses were conducted in a blinded manner to minimize potential observer bias. Behavioral tests were performed prior to TBI induction (baseline), immediately after injury, and weekly thereafter to assess longitudinal functional recovery. Motor and sensorimotor performance was evaluated using the cylinder test, grid-walking assay, and pasta-handling test (Figure 8c).^83^

Forelimb usage asymmetry was assessed using the cylinder test, which measures limb preference during vertical exploration and serves as an indicator of unilateral cortical injury. Following TBI, animals exhibited a pronounced shift toward left forelimb dominance, particularly in the control group, reflecting impaired motor coordination (Figure 8d). In contrast, mice treated with GNP@PMNO under HFMF stimulation showed significantly improved bilateral forelimb use by week four, indicating effective motor function recovery. Motor coordination and limb function were further evaluated using the grid-walking and pasta-handling tests. In the grid-walking assay, TBI induced a sustained increase in hindlimb placement errors, particularly in the untreated control group, indicating persistent motor coordination deficits (Figure 8e). In contrast, mice treated with GNP@PMNO under HFMF stimulation exhibited a significant reduction in foot placement errors by week four, reflecting improved motor recovery and functional neural integration. Fine motor function was assessed using the pasta-handling test. Following TBI, untreated mice exhibited a marked reduction in pasta consumption, indicative of impaired forelimb dexterity (Figure 8f). In contrast, the GNP@PMNO + HFMF group consumed significantly longer pasta segments, reflecting substantial recovery of fine motor control. Together, these findings demonstrate that GNP@PMNO combined with HFMF stimulation effectively restores both gross and fine motor functions after brain injury.

## 3 Conclusion

In this work, we report a multifunctional nanotherapeutic platform composed of platelet membrane–camouflaged gold nanoparticles loaded with nitric oxide (GNP@PMNO) for the treatment of TBI. The platelet membrane coating endows the nanoparticles with immune evasion and injury-site targeting capabilities, enabling efficient accumulation at lesion sites. Under HFMF stimulation, the GNP core functions as a brain-electronic antenna (BEA), transducing external electromagnetic signals into localized bioelectric cues that enhance neuronal activity, while concurrently acting as a miniaturized chemo-electrical reactor to trigger on-demand release of platelet-derived NO. This coupled electrochemical action modulates the inflammatory microenvironment, promotes angiogenesis, and facilitates neural tissue repair. Comprehensive physicochemical characterization confirmed the structural integrity and stability of GNP@PMNO, and HFMF-triggered NO release was validated by Griess assay. *In vitro* studies demonstrated excellent cytocompatibility and enhanced neural stem cell proliferation and neuronal differentiation, whereas *in vivo* administration significantly promoted angiogenesis and neurofilament regeneration while attenuating glial scarring and neuroinflammation in the peri-injury region. Consistently, behavioral assessments revealed substantial recovery of both gross and fine motor functions. This platelet-inspired, electromagnetically responsive nanoplatform establishes a BEA-enabled, miniaturized chemo-electrical reactor as a minimally invasive and targeted therapeutic paradigm, offering strong potential for advancing interventions in TBI and related neurotraumatic disorders.

## 4 Experimental Section

### 4.1 Synthesis of Gold Nanoparticles (GNPs)

We adopted a one-step synthesis method, referencing Srikanth Pedireddy’s approach, to synthesize hollow gold nanoparticles in the presence of silver ions, using HAuCl_4_ as the precursor, hydroquinone as the reducing agent, and PVP as the surface-reducing agent. To control the size of the gold nanoparticles, the synthesis method involved sequentially adding solutions of different concentrations (7, 14, and 28 mM) of 5.7 mL hydroquinone, 2.16 mL 10 mM silver nitrate, and 3.6 mL of 40 mM HAuCl_4_ to the 162 mL of 90 mM PVP solution, with thorough stirring at room temperature. When the solution changed color from colorless to light green and then to dark red, it was left to stand for 3 h. Subsequently, concentrated NH_4_OH was used to remove the free AgCl generated during the reaction, and purification was achieved by repeated centrifugation at 6000 rpm for 10 min.

### 4.2 Synthesis of Platelet Membrane-Nitric Oxide (PMNO)

First, isolate platelets from the whole blood of mice. Collect 1 ml of whole blood from mice via cardiac puncture, and centrifuge at 800 g for 15 min. Carefully collect the upper layer of platelet-rich plasma, and centrifuge at 2000*g* for 15 min to remove the supernatant. Resuspend the pellet in PBS solution. Obtain platelet membranes through repeated freeze-thaw cycles by freezing and thawing the platelets 5 times from room temperature to −80°C. Then, centrifuge at 2000*g* for 20 min to obtain the platelet membranes. It is known that the membrane proteins on platelets contain cysteine, which has an -SH functional group. To attach NO groups to the membrane proteins of platelets, we used a method similar to the synthesis of GSNO from GSH and NaNO_2_. In the resuspended PM solution, we added pH 4 HCl solution and then slowly added NaNO_2_ in an ice bath. After stirring for 40 min, we obtained PMNO by centrifugation at 2000*g*, and washed it three times with water. By treating the PMNO solution and GNP with ultrasound for 1 h, we obtained GNP@PMNO.

### 4.3 Characterizations of GNP@PMNO

The ζ potential of the nanoparticles was measured by dynamic light scattering (DLS; Nano-ZS, Malvern Instruments). All samples were dispersed in deionized water and analyzed in glass cuvettes. The morphology and structural features of the nanoparticles were characterized using field-emission scanning electron microscopy (FE-SEM; JSM-7000F, JEOL, Japan) and transmission electron microscopy (TEM; JEM-2100, JEOL, Japan). Elemental composition and spatial distribution were further analyzed by energy-dispersive X-ray spectroscopy (EDS) coupled with TEM. For SEM analysis, samples were drop-cast onto silicon wafers, dried at room temperature, and sputter-coated with an ultrathin platinum layer to enhance surface conductivity and imaging contrast. For TEM analysis, nanoparticle suspensions were deposited onto copper grids and dried under ambient conditions. Images were acquired from multiple regions to obtain representative morphological and lattice-resolved information. Structural characteristics and phase composition were examined by X-ray diffraction (XRD; APEX DUO, Bruker). Surface chemical states were analyzed using high-resolution X-ray photoelectron spectroscopy (XPS; PHI Quantera II, ULVAC-PHI), while functional group composition was characterized by Fourier-transform infrared spectroscopy (FT-IR; Vertex 80v, Bruker). The magnetoelectric properties of the nanoparticles were evaluated by recording current–time (I–T) curves using amperometric measurements (Dawson), allowing assessment of conductivity and electrically responsive behavior under stimulation.

### 4.4 HFMF Setup and Exposure Conditions

The electrical responsiveness of GNPs and GNP@PMNO under HFMF stimulation was evaluated by monitoring current–time (I–T) behavior. For device measurements, nanoparticles were deposited onto the center of fluorine-doped tin oxide (FTO) substrates, with tin tape used to cover both sides of the FTO as electrodes. During HFMF exposure, the distance between the FTO substrate and the center of the magnetic coil was fixed at 1.5 cm. For cellular and animal experiments, culture dishes and animals were positioned at the same distance from the coil center to ensure consistent field exposure across all assays. The applied HFMF parameters were as follows: field intensity of 2.8 kW and frequency of 1 MHz, with an exposure duration of 3 min for single-stimulation experiments. For pulsed stimulation studies, an on/off switching mode of 5 s on/5 s off (2.8 kW, 1 MHz) was employed.

### 4.5 NO Release assay

Nitric oxide (NO) release was quantified by measuring nitrite concentration in aqueous media using the Griess reaction. Briefly, the Griess reagent was prepared by dissolving 40 mg of Griess powder in 1 mL of deionized water. For NO release measurements, 100 mg of GNP@PMNO particles were dispersed in 1 mL of deionized water. At predetermined time points, 500 μL of the sample solution was mixed with 500 μL of the Griess reagent and incubated at room temperature for 15 min. The absorbance at 540 nm was recorded using a UV–vis spectrophotometer (SP-8001, Metertech), and nitrite concentrations were determined based on a standard calibration curve. For HFMF–treated groups, nanoparticle suspensions containing GNP@PMNO or PMNO were exposed to HFMF for varying durations prior to analysis. Nitrite levels were subsequently quantified using the same Griess assay protocol. To determine the total NO content, GNP@PMNO was heated to 70 °C to cleave S–NO bonds and induce complete NO release. After equilibration for 24 h, the released NO was defined as 100%. The cumulative NO release was calculated according to the following equation:

$$\mathrm{cumulative NO release}=\left(\frac{\mathrm{released NO}}{\mathrm{total NO}}\right)\times 100\%$$

### 4.6 Cellular Membrane Leakage under HFMF Irradiation

To evaluate DAPI membrane penetration efficiency, a modified membrane disruption assay was performed. NIH-3T3 cells (1 × 10^5^ cells/mL) were first seeded into 6-well plates and cultured at 37 °C for 24 h. The culture medium was then replaced with fresh medium containing the designated particle formulations, followed by an additional 30 min incubation. After treatment, the particle-containing medium was removed, and the cells were gently rinsed with phosphate-buffered saline (PBS) 1–2 times to remove unbound particles. Subsequently, the cells were incubated with DAPI solution (1 μg/mL) for 30 min to assess membrane permeability, followed by PBS washing to eliminate excess dye. For the HFMF-treated groups, cells were exposed to a high-frequency magnetic field (1 MHz, 2.8 kW) for 3 min immediately after particle administration. Following all treatments, cell viability was evaluated using the LIVE/DEAD Viability/Cytotoxicity Kit (Invitrogen, L3224), and fluorescence images were acquired using confocal laser scanning microscopy.

### 4.7 *In Vitro* Cell Culture and Cytotoxicity

NIH-3T3 cells were cultured in a 37 °C incubator with 5% CO_2_ using DMEM supplemented with 10% fetal bovine serum (FBS) and 1% penicillin. The culture medium was refreshed every 3 days. When subculturing the cells, the spent DMEM was aspirated from the 10 cm culture dish. The cells were gently washed with warm PBS, and then 1 ml of warm 0.25% trypsin-EDTA was added to the dish. The dish was placed in a 37°C incubator with 5% CO_2_ for 3 min to allow trypsin digestion. To stop the trypsin reaction, 2 ml of DMEM was added to the dish. The cells were then centrifuged at 800 rpm for 5 min. The cell count was determined using a cell counter and trypan blue staining. Cytotoxicity was evaluated using NIH-3T3 fibroblasts cultured in 96-well plates at a density of 1 × 10^4^ cells per well in 100 μL of complete culture medium. After 24 h of cell attachment, the materials were incubated in culture medium at 37 °C for 24 h to obtain material extracts. The supernatants were then collected and diluted to the desired concentrations to serve as the testing media. Cells were subsequently incubated with the extracted media for an additional 24 h. Following exposure, 10 μL of PrestoBlue reagent was added to each well and incubated for 10 min at 37 °C. Fluorescence intensity was measured using a microplate reader (Synergy HT Multi-Detection Microplate Reader, BioTek Instruments, Inc., USA) at an excitation wavelength of 540 nm. Cell viability was calculated relative to untreated control cells and expressed as a percentage of the control.

### 4.8 *In Vitro* Cell Uptake

NIH-3T3 cells were seeded in confocal culture dishes at a density of 1 × 10^4^ cells/mL and incubated at 37 °C. Cy5.5-labeled GNP and GNP@PMNO were added to the culture medium to ensure uniform distribution. On day 3, cell nuclei were stained with DAPI, and the cytoskeleton was labeled with F-actin. PBS washes were performed between staining steps to ensure clarity. Samples were imaged using a laser scanning confocal microscope (Zeiss LSM-780), with excitation/emission settings optimized for Cy5.5, DAPI, and F-actin. Image analysis was performed to evaluate nanoparticle localization and interaction with cellular structures.

### 4.9 *In Vitro* Lysosomal Escape

To evaluate the lysosomal escape behavior of the nanoparticles, LysoTracker staining was performed. NIH-3T3 cells were seeded in 3 cm confocal dishes at a density of 5 × 10^5^ cells mL^–1^ and cultured for 24 h to allow cell attachment. Nanoparticles were then added and incubated with the cells for 4 h. Subsequently, LysoTracker (Invitrogen, L7526) and Hoechst 33342 (Invitrogen, H3570) were diluted 1:1000 in PBS to prepare the staining solution, where LysoTracker was used to label acidic lysosomal compartments and Hoechst 33342 was used for nuclear counterstaining. After nanoparticle incubation, cells were washed with PBS to remove uninternalized particles and then incubated with the staining solution for 45 min at 37 °C. Fluorescence imaging was performed using a temperature-controlled laser scanning confocal microscope.

### 4.10 Embryonic NSCs Extraction Experiment

Neural stem cells were isolated from embryonic day 16 (ED16) Wistar rat embryos. Following euthanasia, pregnant rats were transferred to a laminar flow hood. Embryos were excised from the uterus, washed with Hank’s Balanced Salt Solution (HBSS), and the right cerebral hemispheres were dissected. Neural tissue was minced and suspended in HBSS. The cell suspension was centrifuged twice at 1000 rpm for 7 min each. Pelleted cells were resuspended in F12/DMEM medium supplemented with 1% penicillin and 1% N-2 supplement, and transferred to 5 mL culture flasks. To support proliferation, 5 mL of neurobasal medium and 10 μL of basic fibroblast growth factor (bFGF) were added. Cells were incubated at 37 °C in a humidified atmosphere with 5% CO_2_. By day 4, neurosphere-like structures were observed in suspension, with occasional adherence to the flask walls. Maintenance medium was refreshed weekly.

## Supplementary Material



## Notes

Surface marker characterization of cell-membrane-cloaked nanoparticles (Figure S1); SEM image and I–T curves of ultrasmall GNPs (Figure S2); cellular membrane disruption under HFMF stimulation (Figure S3); XRD patterns of GNP (Figure S4); stability evaluation of GNP@PMNO under aqueous conditions (Figure S5); the Griess assay detects nitrite (Figure S6); cumulative NO release profiles of GNP@PMNO (Figure S7); cell viability of 3T3 cells after treated by GNP@PMNO (Figure S8); CLSM images showing cellular uptake of GNP and GNP@PMNO (Figure S9); CLSM images showing cellular uptake of GNP@PMNO+HFMF (Figure S10); CLSM images showing colocalization of lysosomes and nanoparticles (Figure S11); CLSM images of *in vitro* NO release detected using DAF-FM (Figure S12); redox-modulating effects associated with the HFMF-triggered NO release (Figure S13); *in vitro* neural differentiation assays (Figure S14); protein microarray analysis elucidating the molecular mechanisms (Figure S15); *in vivo* photothermal safety evaluation and short-term perilesional pathological responses (Figure S16); activation and mobilization of endogenous neural stem cells (Figure S17); CLSM images of peri-lesional brain tissue at 7 days postinjury (Figure S18); *in vivo* protein microarray analysis elucidating the molecular mechanisms (Figure S19) ; the biodistribution of GNP and GNP@PMNO (Figure S20); *in vivo* imaging system (IVIS) analysis in the brain area (Figure S21); H&E staining of brain and major organs (Figure S22); and evaluation of long-term *in vivo* liver toxicity via serum biochemistry analysis (Figure S23) (PDF)
