# Supplementary material for: Nonsurgical Platelet Membrane-Decorated Gold Yarnball Brain Antenna for In Situ Nitric Oxide Generation and Neuronal Regeneration
Source: ACS Nano. 2026 Sep 16;20(38):26235–56. doi: 10.1021/acsnano.6c12030 (PMC13629675; doi:10.1021/acsnano.6c12030)
Supplement: Supplementary file 1 [file nn6c12030_si_001.pdf]

Supporting Information

Nonsurgical Platelet Membrane–Decorated Gold Yarnball Brain Antenna for *In Situ* Nitric Oxide Generation and Neuronal Regeneration

Wan-Chi Pan[1†], *Ming-You Shie*[2,3,4†], *Jia-Yue Xu*[1], *Thi My Hue Huynh*[1], *Hsiu-Ching Liu*[1], *Ngoc-Tri Tran*[1], *Hoi Man Iao*[1], *Hui-Wen Lien*[1,5], *Tsu-Chin Chou*[6], *Wen-Hsuan Chiang*[7], *Shang-Hsiu Hu*[1,6]*

[1] Department of Biomedical Engineering and Environmental Sciences, National Tsing Hua University, Hsinchu, 300044, Taiwan

[2] Department of Biomedical Engineering, China Medical University, Taichung, 406040, Taiwan

[3] Xenotransplantation Translational Research Center, China Medical University Hospital, Taichung, 404327, Taiwan

[4] Department of Bioinformatics and Medical Engineering, Asia University, Taichung, 413305, Taiwan

[5] National Institute of Cancer Research, National Health Research Institutes, Miaoli County, 35053, Taiwan

[6] Institute of Analytical and Environmental Sciences, National Tsing Hua University, Hsinchu, 300044, Taiwan

[7] Department of Chemical Engineering, National Chung Hsing University, Taichung, 402, Taiwan

E-mail: shhu@mx.nthu.edu.tw

Experimental Section

Differentiation of NSCs experiment

To promote NSC adhesion, coverslips were first cleaned with ethanol, exposed to UV light for 15 minutes, and coated with 100 μL of poly-L-lysine (PLL). Coverslips were incubated under UV light for 1 hour, followed by three washes with sterile double-distilled water (ddH_2O), and stored until use. Prior to seeding, coverslips were washed three times with PBS. NSCs were counted using a capture–release method under a microscope. Approximately 20 μL of culture medium was added per well in a 24-well

plate, and ten neurospheres were seeded per well based on calculated cell numbers. After allowing ~8 hours for attachment, GNPs or GNP@PMNO (200 μg/mL) were added to respective wells. Groups requiring electromagnetic stimulation were exposed to a HFMF for 3 minutes daily. NSCs were cultured in differentiation medium consisting of F12/DMEM supplemented with 1% penicillin, 1% N-2, and 0.2% B-27.

After 7 days, cells were fixed with 70% methanol for 5 minutes, washed with PBS, and blocked with 5% BSA for 1 hour at room temperature. Samples were incubated overnight at 4 °C with primary antibodies: mouse anti-GFAP (1:800) and anti-βIII-tubulin (TUJ1, 1:800). After PBS washes, cells were incubated for 1 hour with secondary antibodies: donkey anti-mouse IgG (1:1000) and goat anti-rabbit IgG 488 (1:1000), followed by DAPI counterstaining. All samples were imaged using a laser scanning confocal microscope (ZEISS LSM-780). ImageJ software was used for quantification of differentiation. The proportion of astrocytes and neurons was determined by calculating the intensity of GFAP and TUJ1 staining in the extracellular regions of neurospheres across multiple fields of view.

Serum Biochemistry Analysis

To evaluate potential systemic off-target toxicity and functional liver safety, mouse blood samples were collected via cardiac puncture under anesthesia at 30 days post-treatment. The whole blood was transferred into sterile microcentrifuge tubes without anticoagulants and allowed to clot at room temperature for 45 min. Serum was separated by centrifugation at 3,000 rpm (2,000 × g) for 15 min at 4 °C and stored at −80 °C prior to analysis. Quantitative measurements of serum liver function enzymes, specifically alanine aminotransferase (ALT) and aspartate aminotransferase (AST), were performed using standard biochemical enzymatic assay kits (MAK576, Sigma-Aldrich) according to the manufacturer's instructions. Absorbance values were

measured using a microplate reader, and enzyme activities were calculated and expressed as units per liter (U/L)

Brain collection and immunofluorescence staining

After sacrificing the mice, their brains were collected and fixed in 4% paraformaldehyde solution overnight. Subsequently, the brains were dehydrated by immersion in sucrose solutions of increasing concentrations: 10% for 10 minutes, 15% for 10 minutes, 20% for 30 minutes, and 30% overnight. The brains were then embedded in optimal cutting temperature compound (OCT) and sectioned into slices approximately 15 μm thick using a cryostat at -20°C for immunofluorescence analysis.

The slices were first fixed in methanol at -20°C for 10 minutes and then washed three times with PBS. To reduce nonspecific binding of antibodies, the slices were immersed in a 5% BSA solution at room temperature for 1 hour. Subsequently, the slices were incubated overnight at 4°C with specific antibodies: (1) CD31 for vascular cells (rat, dilution 1:800), (2) NF200 for regenerating nerve fibers (rabbit, dilution 1:800), (3) GFAP for astrocytes (rabbit, dilution 1:800), and (4) Iba1 for microglia (goat, dilution 1:800).

One day later, the slices were washed three times with PBS and then incubated with secondary antibodies at room temperature for one hour: (1) Goat Anti-Rat IgG (H+L) Alexa 488 (dilution 1:1000), (2) Goat Anti-Rabbit IgG (H+L) Cy3 (dilution 1:1000), (3) Donkey Anti-Rabbit IgG (H+L) Alexa 647 (dilution 1:1000), and (4) Rabbit Anti-Goat IgG (H+L) Alexa 488 (dilution 1:1000). Subsequently, the slices were washed three times with PBS and then mounted with DAPI. The morphology of stained slices was observed using laser scanning confocal microscopy. Image J software was used to analyze the intensity and percentage of differentiated cells.

Animal behavior experiment

Three different behavioral tests were utilized: the cylinder test, the grid test, and pasta test. These tests were conducted on mice before injury and at weeks 0, 1, 2, 3, and 4 post-injury. (1) Cylinder Test: The cylinder test is employed to assess the forelimb dexterity of mice. Using an acrylic glass cylinder, each mouse is individually placed inside to observe its forelimb movements. The number of contacts between the forelimbs and the cylinder is recorded within one minute, and the asymmetry between the left and right forelimbs is measured. The following formula is used to calculate the asymmetry index: [R + (R+L)] - [L+ (R+L)] / [R + (R+L)] + [L+ (R+L)]. The diameter of the acrylic glass cylinder is 10 cm, and the height is 15 cm. (2) Grid Test: The purpose of the grid test is to evaluate the flexibility of the hind limbs of mice. This test utilizes a grid area measuring 12 cm in length, 36 cm in width, and 10 cm in height. Mice are placed individually on the grid, and their movements on the grid are observed. When a mouse moves on an obstacle, it may miss a foothold, which is recorded. The following formula is then used to calculate and express it as a percentage: (number of steps / total steps) × 100. (3) Pasta Test: The objective of the pasta test is to assess the controllability of the forelimbs of mice. Spaghetti with a diameter of 1 mm is cut into lengths of 2.6 cm. During the test, the length of spaghetti each mouse can eat within three minutes is recorded as a measure of its forelimb control ability.

TBI model and brain collection

A TBI mouse model was established in 7-week-old female C57BL/6 mice according to a previously published protocol developed by our group. All animal experiments were performed following the protocols evaluated and approved by the the Institutional Animal Care and Use Committee (IACUC) of National Tsing Hua University (Approval No. 112006). Briefly, mice were euthanized, and a circular cranial window was created using a high-speed drill. A flat-ended biopsy punch with a diameter of 2 mm was then used to generate a standardized cortical lesion by removing

brain tissue to a depth of 1.5 mm. Immediately following injury induction, nanoparticles were administered via intravenous tail vein injection, with each mouse receiving 100 μL of solution at a concentration of 2 mg mL^{-1}. Control animals received an equivalent volume of phosphate-buffered saline (PBS). For groups requiring magnetic stimulation, animals were subjected to high-frequency magnetic field (HFMF) treatment for 3 min per day until sacrifice.

Statistical Analysis

Statistical analyses were performed using GraphPad Prism (version 9.0), based on data from at least three independent experiments. Error bars represent the standard deviation (SD). One-way analysis of variance (ANOVA) followed by Tukey's multiple comparisons test was used to determine statistical significance, as indicated in the figure legends. Statistical significance was defined as $p < 0.05$ (*), $p < 0.01$ (**), $p < 0.001$ (***), and $p < 0.0001$ (****).

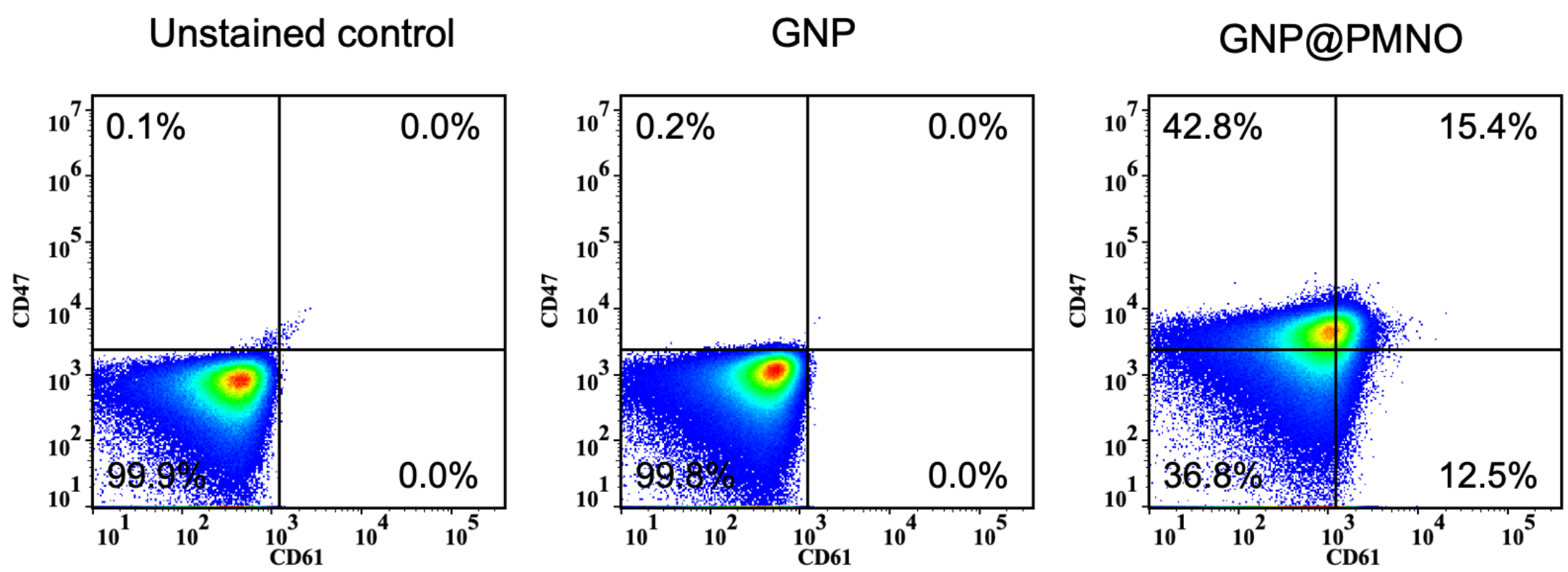

Figure S1. Surface marker characterization of cell-membrane-cloaked nanoparticles. Representative dual-parameter flow cytometry dot plots validating the surface presentation and topological preservation of key donor membrane proteins on the biomimetic vectors. Corresponding gating percentages for each quadrant are indicated within the panels.

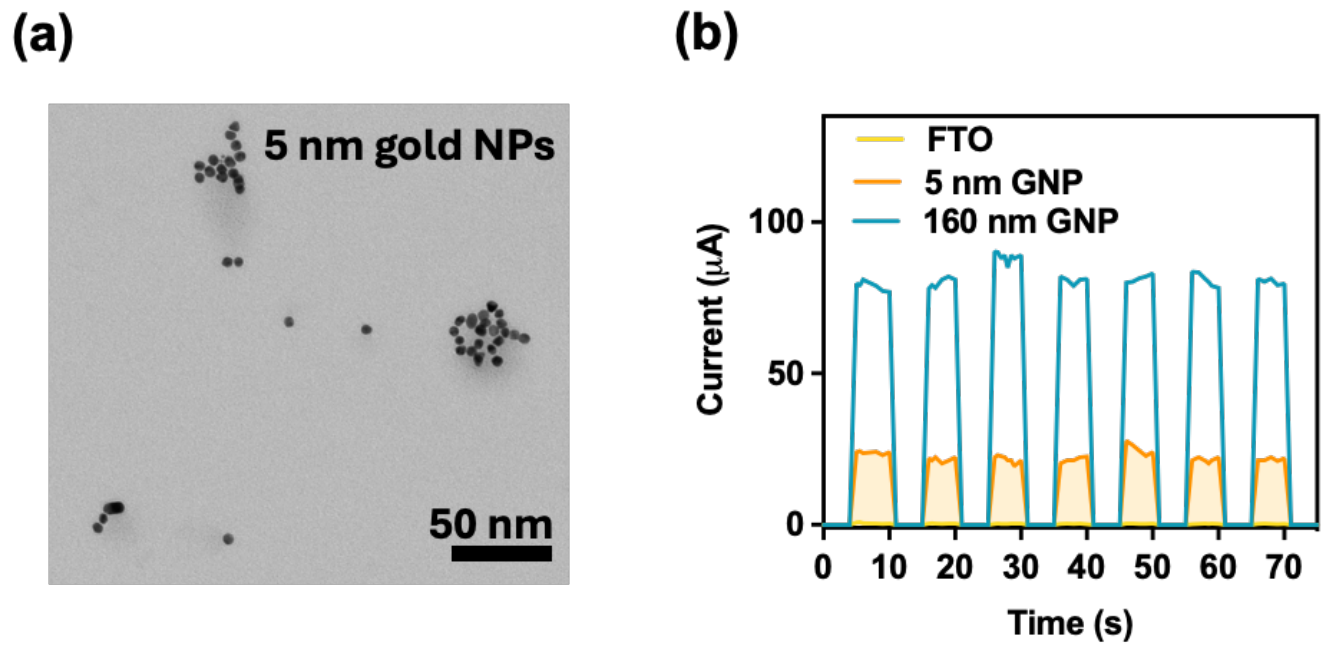

Figure S2. (a) SEM image of ultra-small GNPs with an average diameter of ~5 nm. (b) I–T curves of 5 nm and 160 nm GNPs under HFMF stimulation, demonstrating their size-dependent electrical response capabilities.

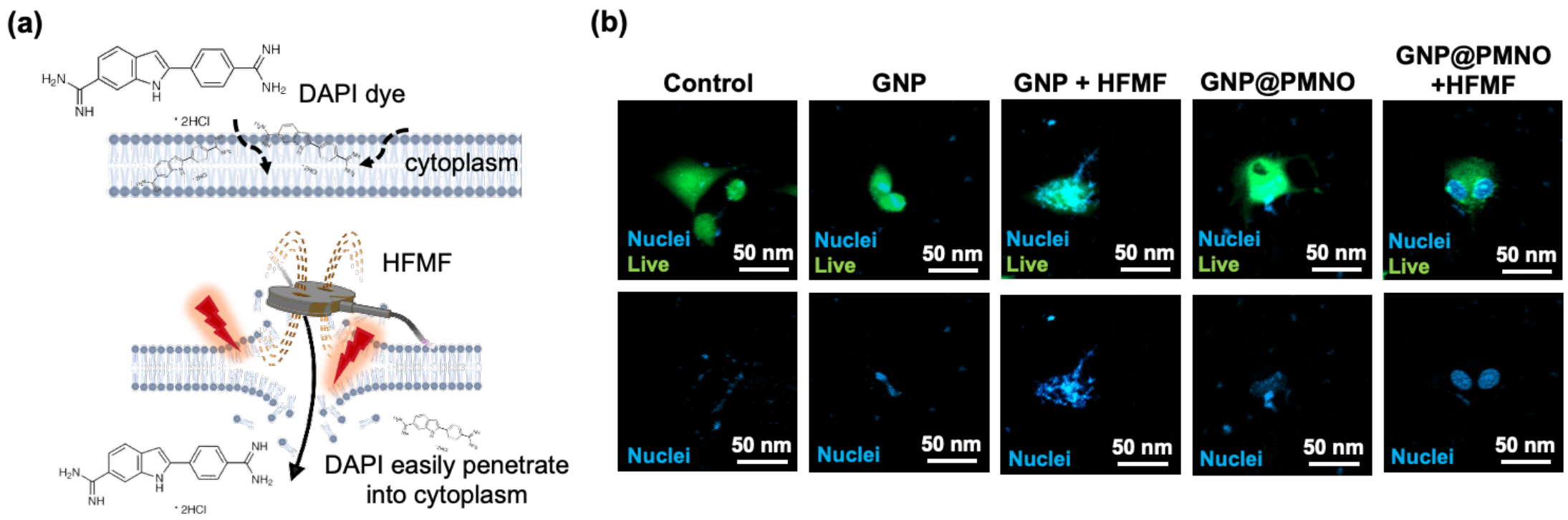

Figure S3. Cellular membrane disruption under HFMF stimulation. (a) Schematic illustration of HFMF-induced cellular membrane leakage. (b) Representative fluorescence images showing membrane disruption and enhanced membrane permeability following HFMF irradiation. DAPI labels cell nuclei, while calcein-AM stains viable cells.

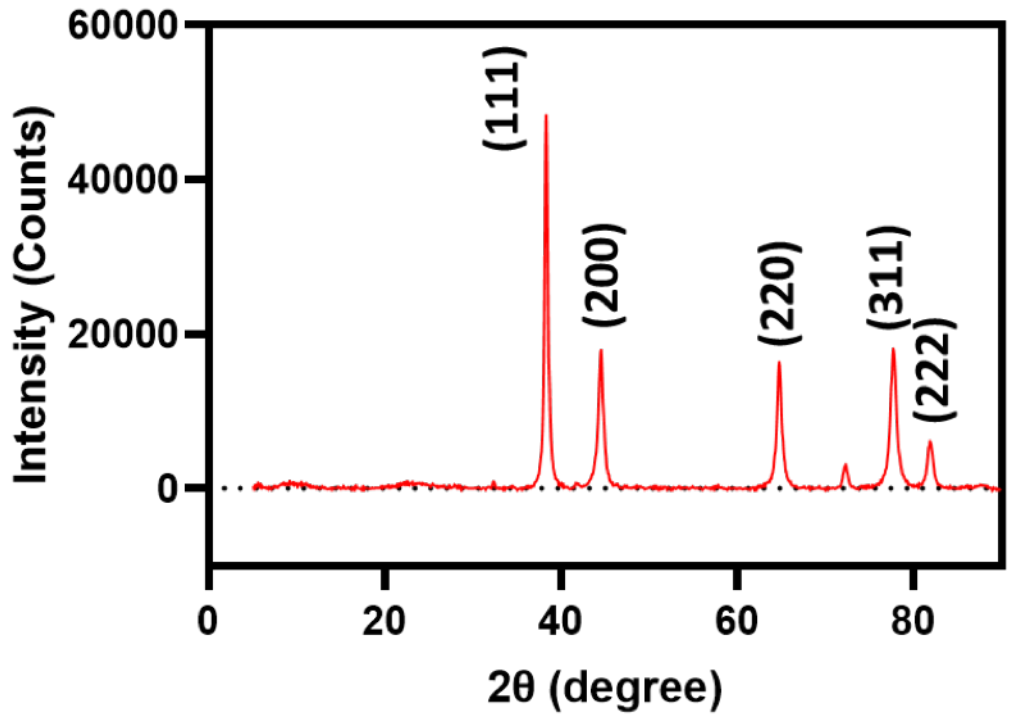

Figure S4. XRD patterns of GNP.

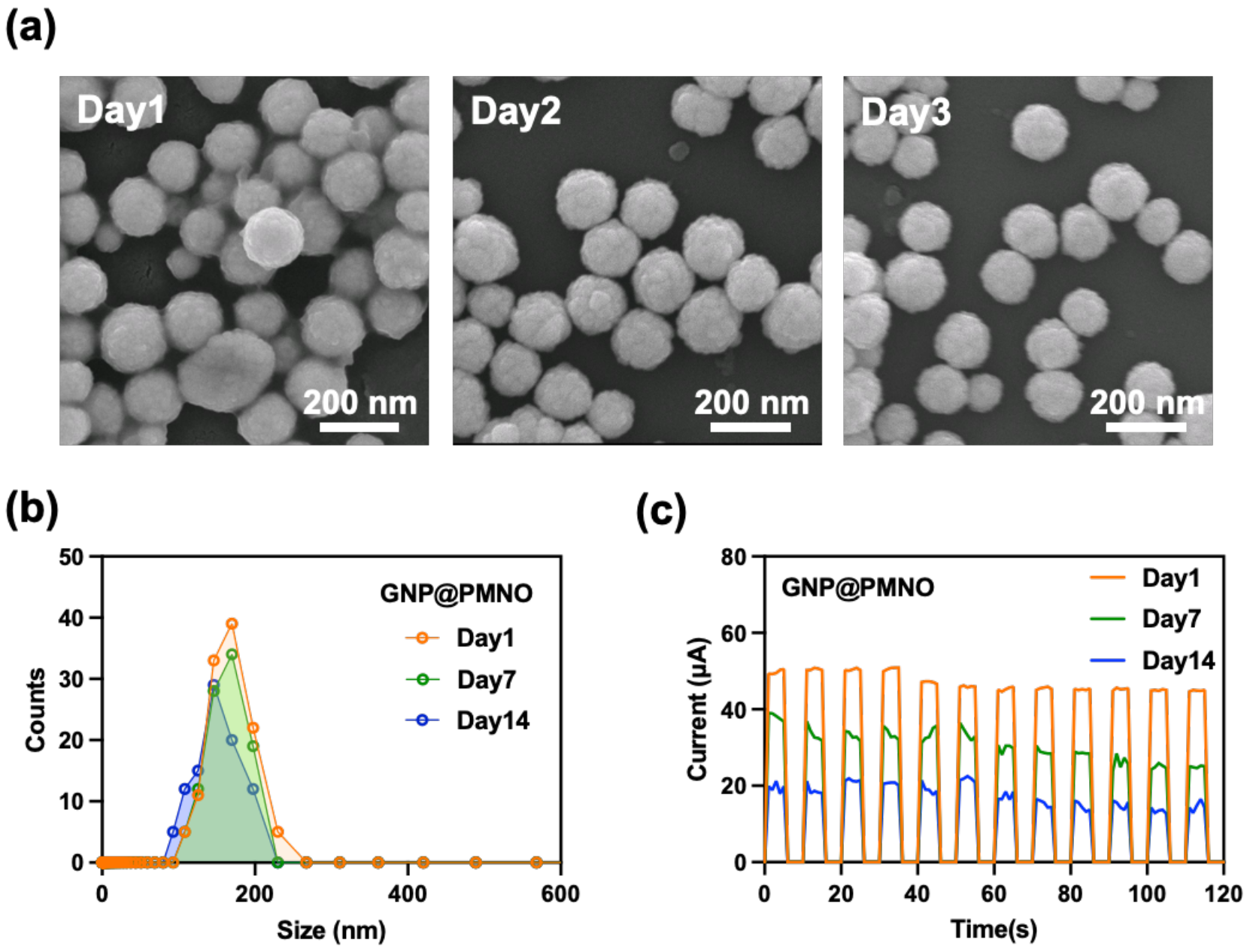

Figure S5. Stability evaluation of GNP@PMNO under aqueous conditions. (a) SEM images of GNP@PMNO after storage in PBS solution. (b) Hydrodynamic size distribution of GNP@PMNO measured by DLS during the storage period. (c) Electrical responsiveness of GNP@PMNO under repeated HFMF stimulation, evaluated by current–time (I–T) measurements.

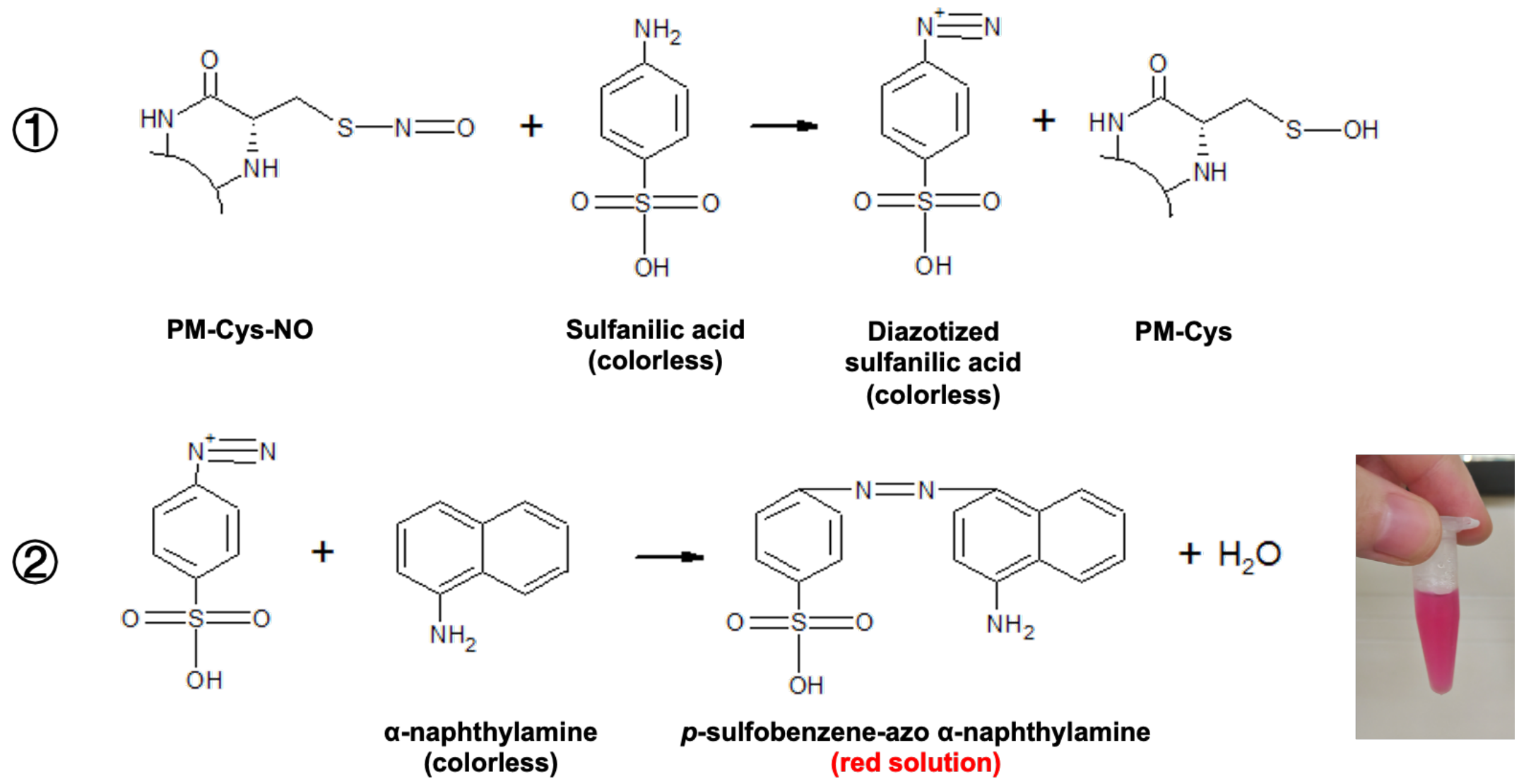

Figure S6. The Griess assay detects nitrite (NO_2^-) via diazotization and subsequent azo-coupling reactions, producing a pink-colored azo dye.

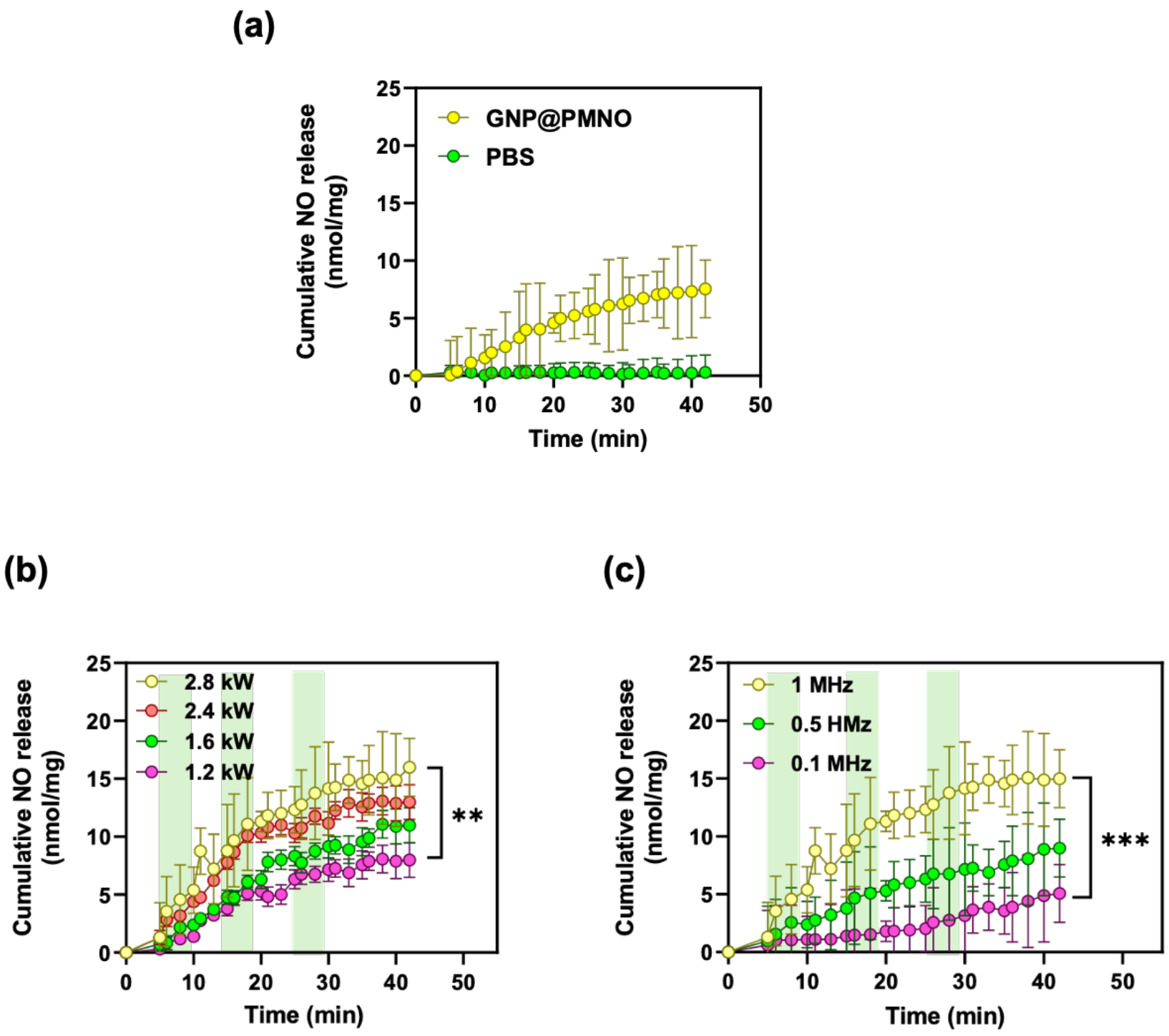

Figure S7. Cumulative NO release profiles of GNP@PMNO measured by DAF-FM fluorescence under different stimulation conditions: without HFMF irradiation (a), under varying HFMF intensities (b), and under varying HFMF frequencies (c). Data are

presented as mean ± s.d. (n = 5). Statistical significance was determined by one-way ANOVA followed by Tukey's multiple-comparison test (**$p < 0.01$, ***$p < 0.001$).

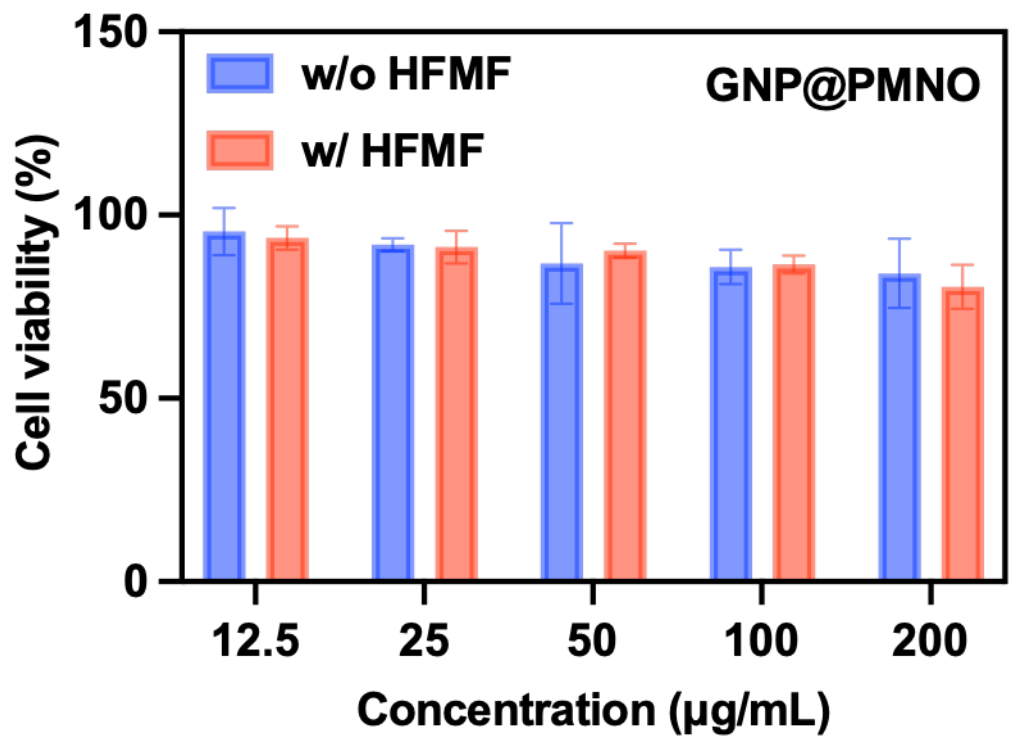

Figure S8. Cell viability of 3T3 cells after treated by GNP@PMNO with HFMF treatment.

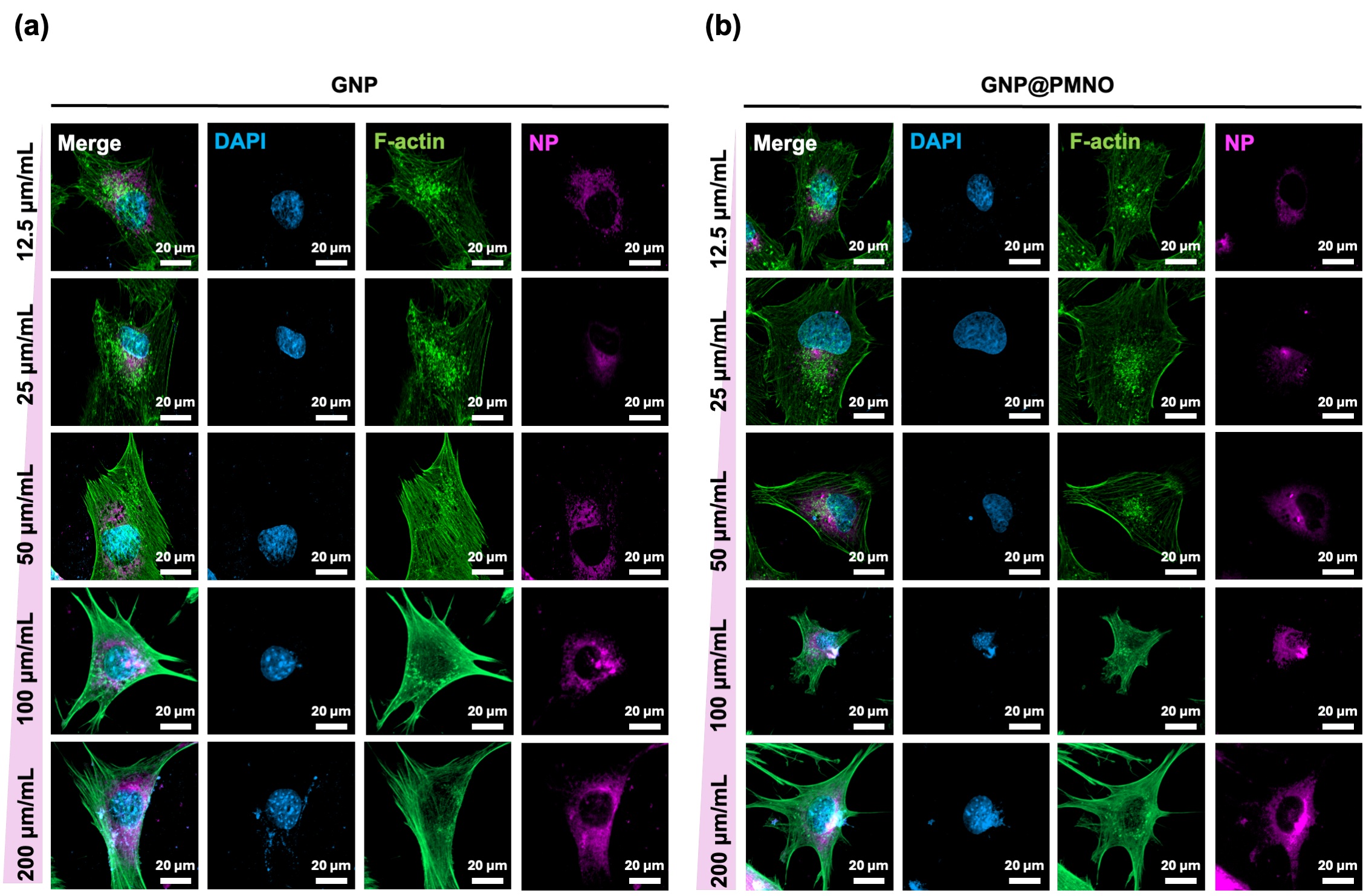

Figure S9. CLSM images showing cellular uptake of GNP and GNP@PMNO at different concentrations. Blue fluorescence indicates cell nuclei stained with DAPI, green fluorescence represents the cytoskeleton stained with F-actin, and purple fluorescence denotes the nanoparticles.

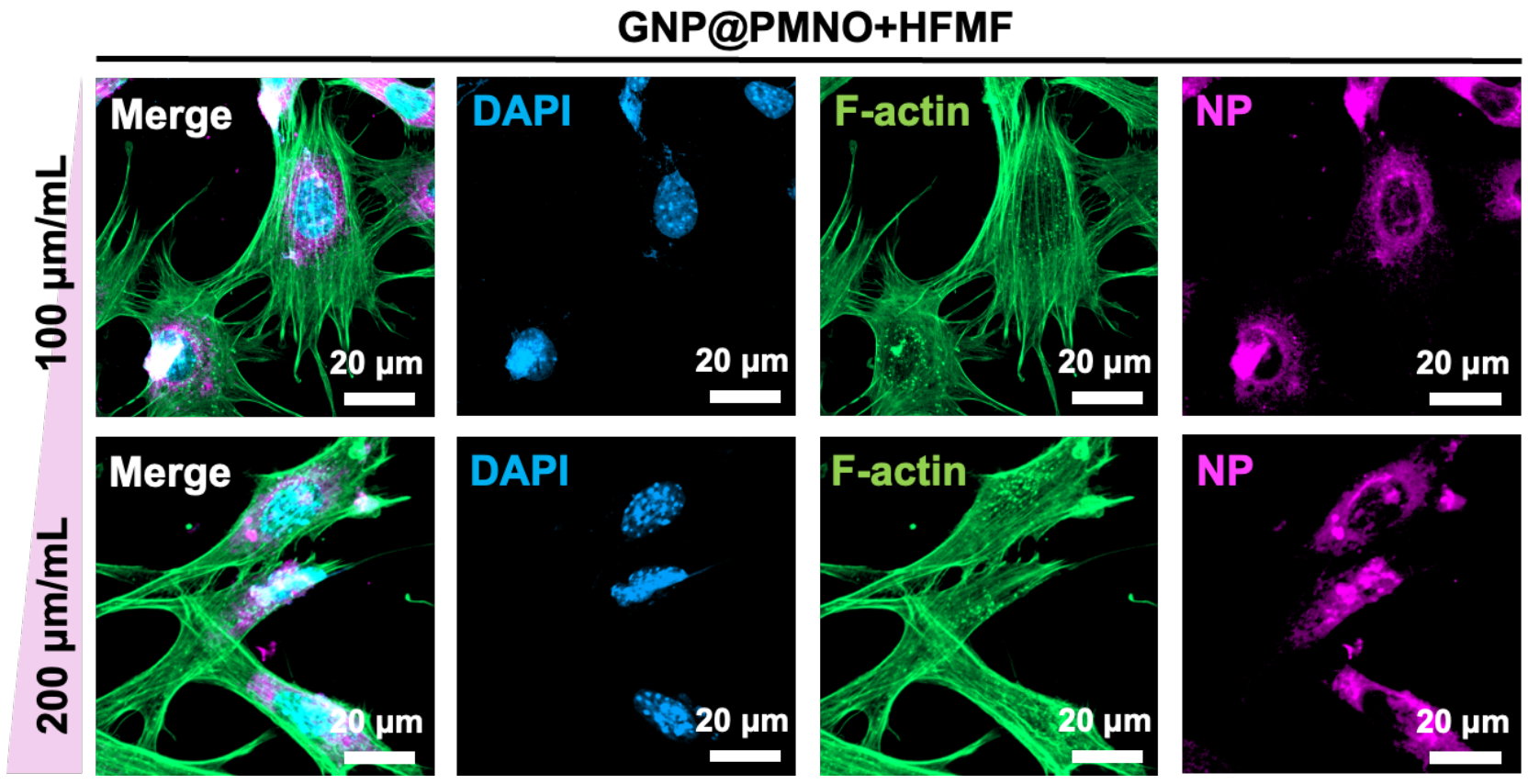

Figure S10. CLSM images showing cellular uptake of GNP@PMNO+HFMF. Blue fluorescence indicates cell nuclei stained with DAPI, green fluorescence represents the cytoskeleton stained with F-actin, and purple fluorescence presents the nanoparticles.

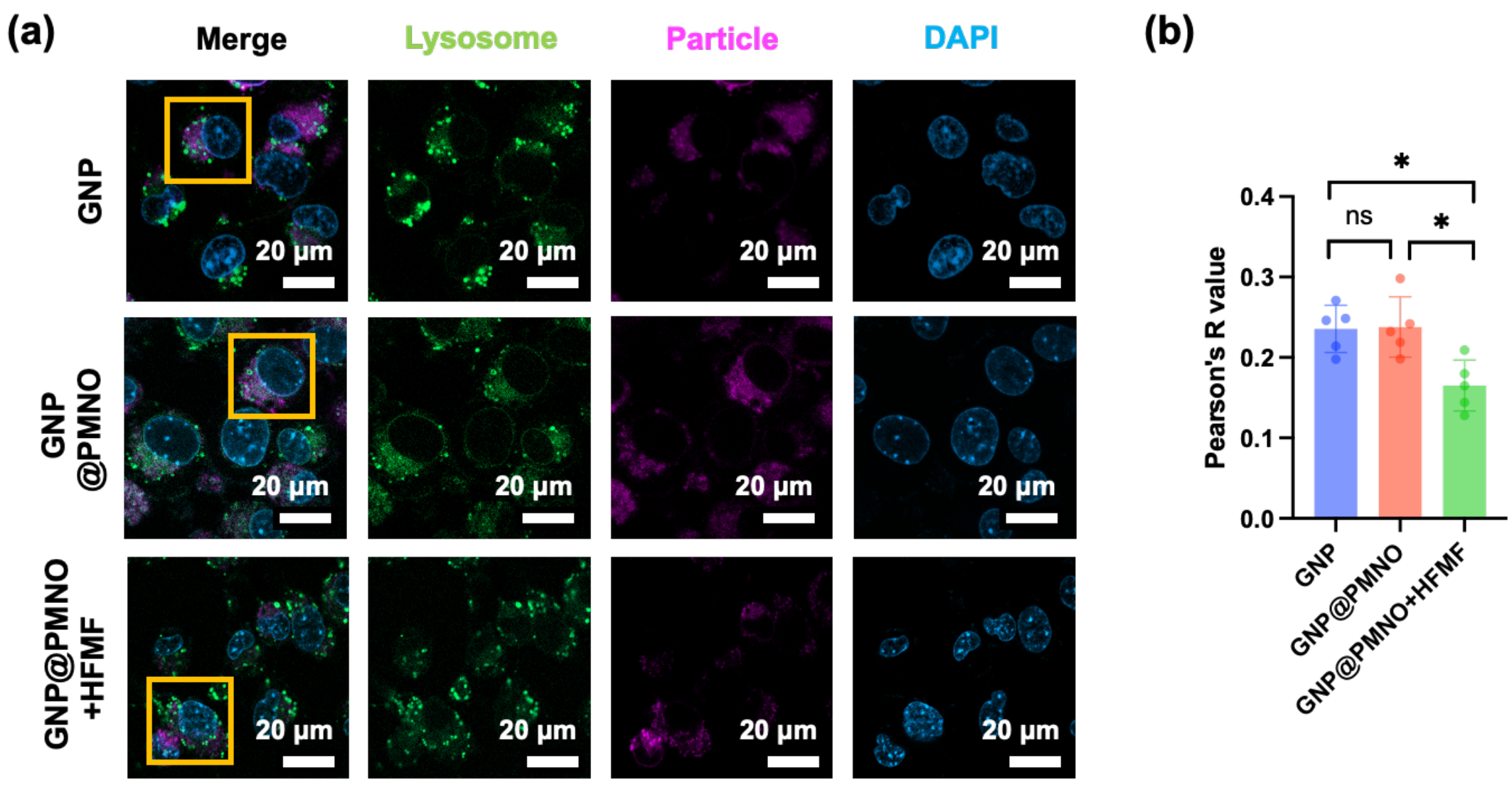

Figure S11. (a) Confocal laser scanning microscopy (CLSM) images showing co-localization of lysosomes (LysoTracker, green) and nanoparticles (red). (b) Quantitative analysis of co-localization based on Pearson's correlation coefficient. (n = 10, mean ± s.d.; $^{**}p < 0.01$, one-way ANOVA with Tukey's multiple comparison test).

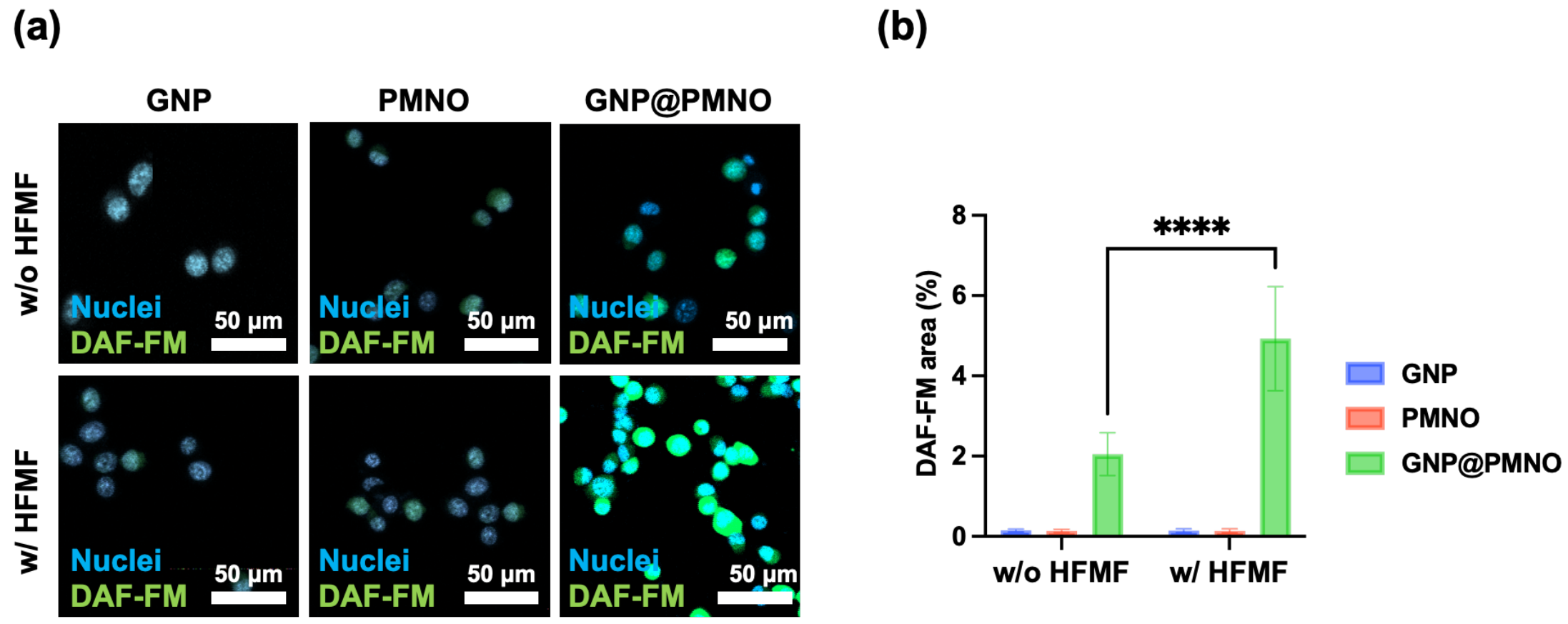

Figure S12. (a) CLSM images showing *in vitro* NO release detected using DAF-FM. (b) Quantification of DAF-FM fluorescence intensity in each group with or without HFMF stimulation.

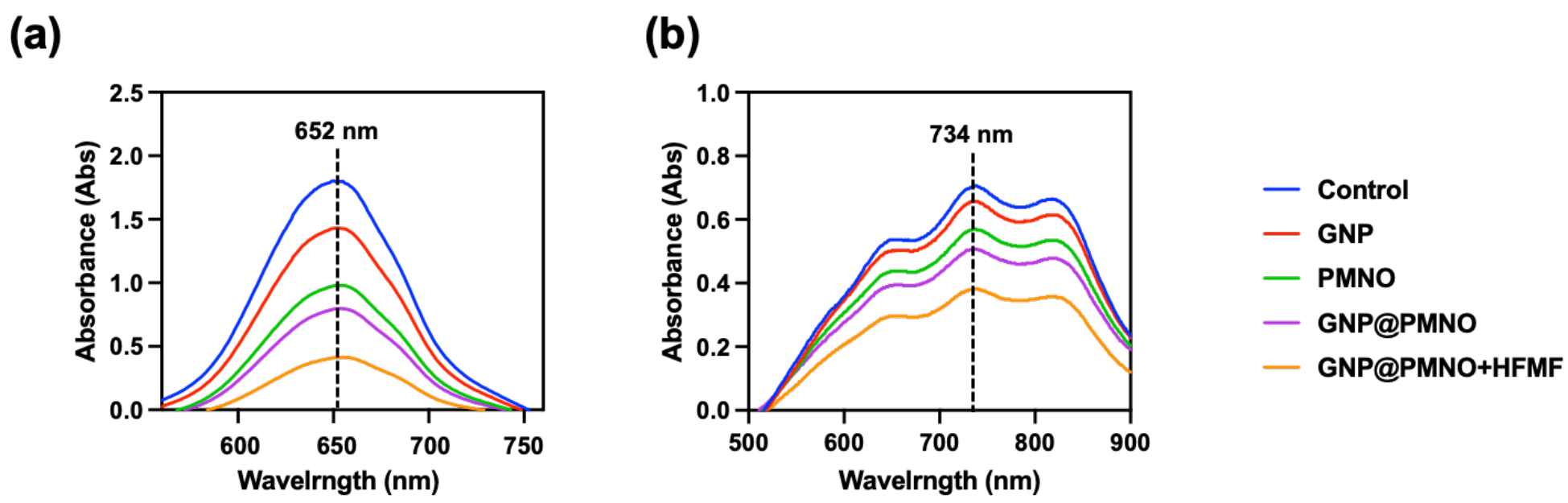

Figure S13. Redox-modulating effects associated with the HFMF-triggered NO release capability of the GNP@PMNO system. (a) TMB assay showing suppression of oxidative chromogenic reaction. (b) ABTS radical-scavenging assay demonstrating decreased absorbance of $ABTS^{\cdot+}$ at 734 nm.

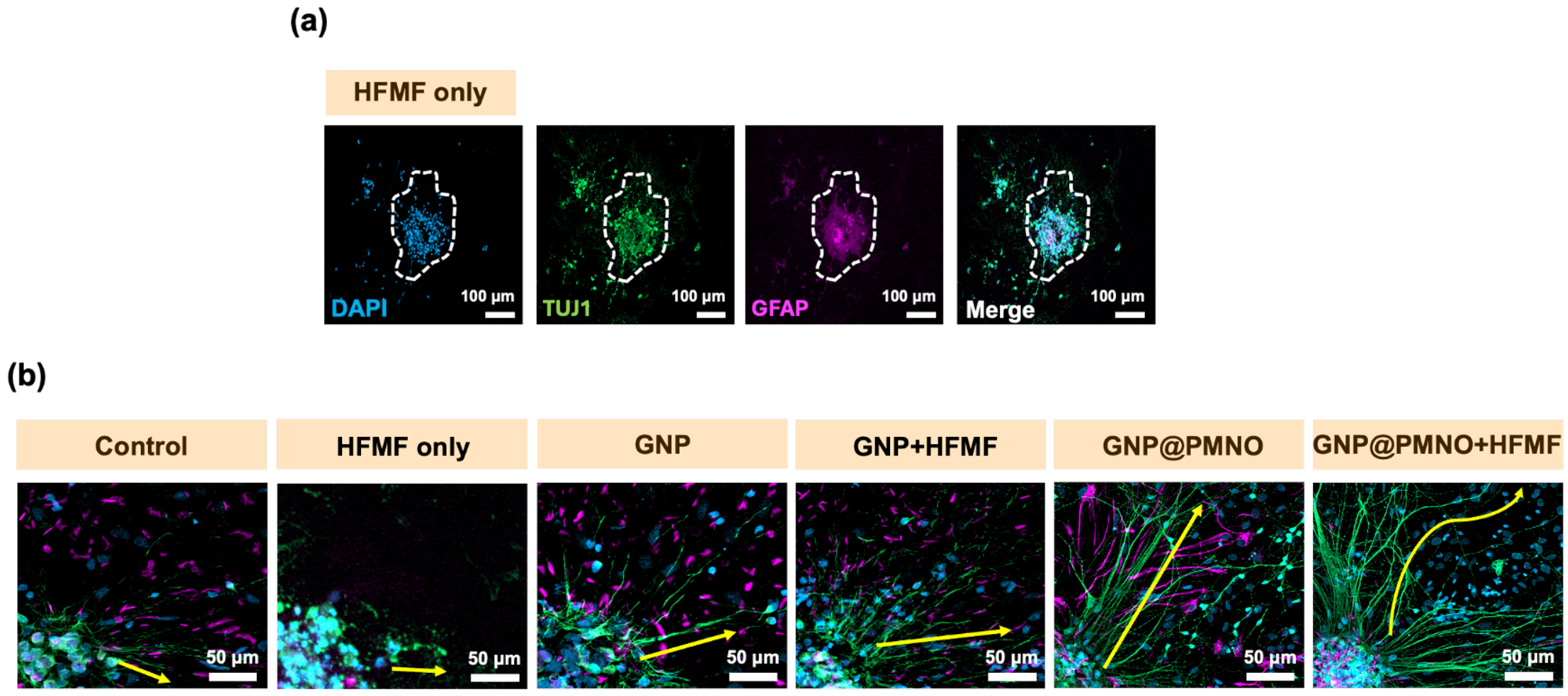

Figure S14. ***In vitro*** **neural differentiation assays.** (a) Representative CLSM images of the HFMF-only group showing TUJ-1-positive neurons (green), GFAP-positive astrocytes (purple), and DAPI-stained nuclei (blue). High-magnification CLSM images illustrating neuronal outgrowth under different treatment conditions, including control, HFMF only, GNP, GNP + HFMF, GNP@PMNO, and GNP@PMNO + HFMF groups.

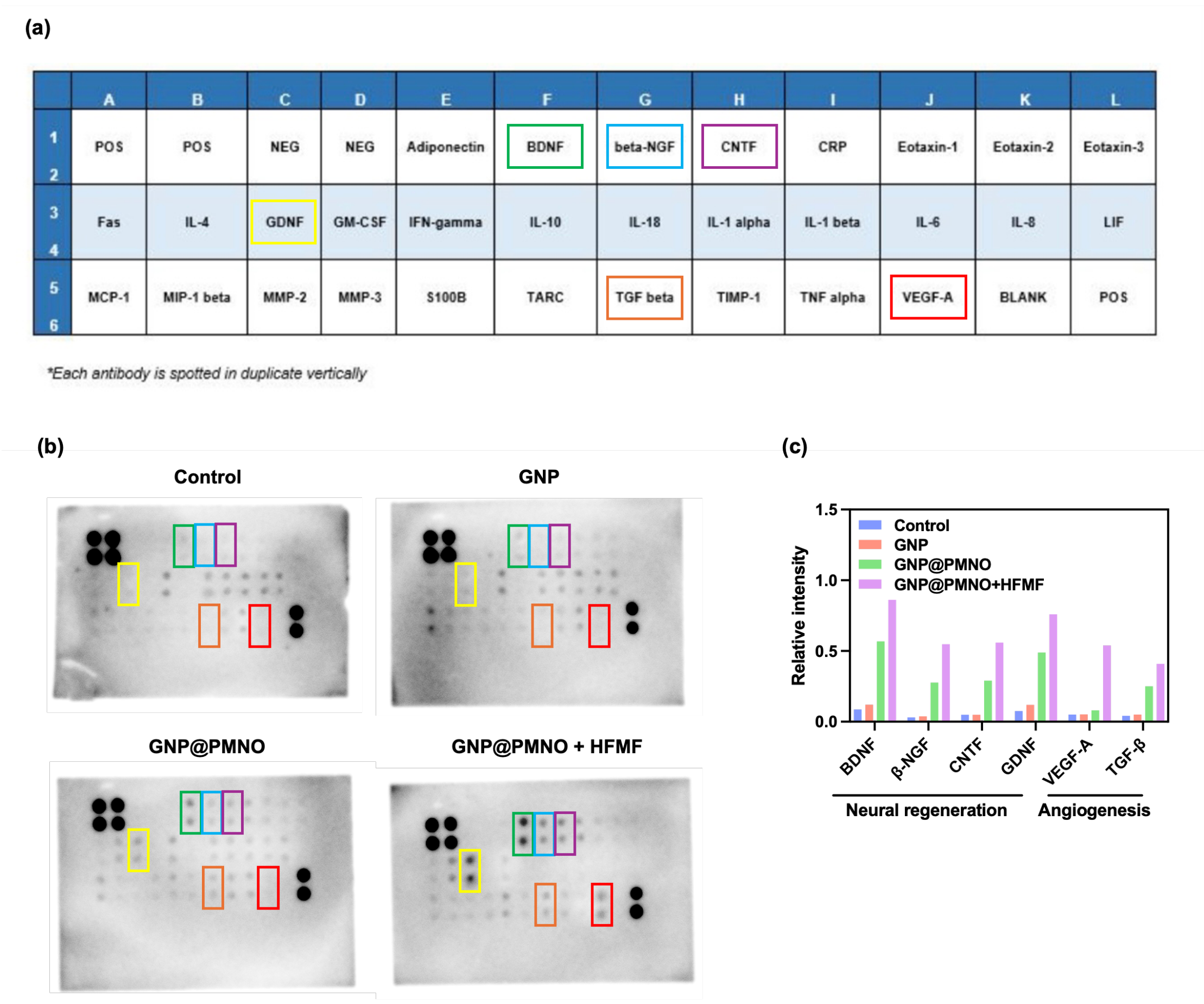

Figure S15. Protein microarray analysis elucidating the molecular mechanisms underlying GNP@PMNO-mediated neural regeneration under HFMF stimulation. (a) Schematic illustration of the antibody spotting layout used in the protein microarray, highlighting cytokines, neurotrophic factors, and angiogenesis-related proteins. (b) Representative protein microarray images showing the expression profiles of cytokines and neuroregenerative factors under different treatment conditions. (c) Quantitative analysis of selected cytokines, neurotrophic factors, and angiogenesis-related proteins. Notably, the GNP@PMNO + HFMF group exhibited increased expression of neurotrophic factors, including BDNF, β-NGF, CNTF, and GDNF, as well as angiogenesis-associated factors VEGF-A and TGF-β, indicating the establishment of a neuroprotective and regenerative microenvironment that may facilitate neural repair and functional recovery.

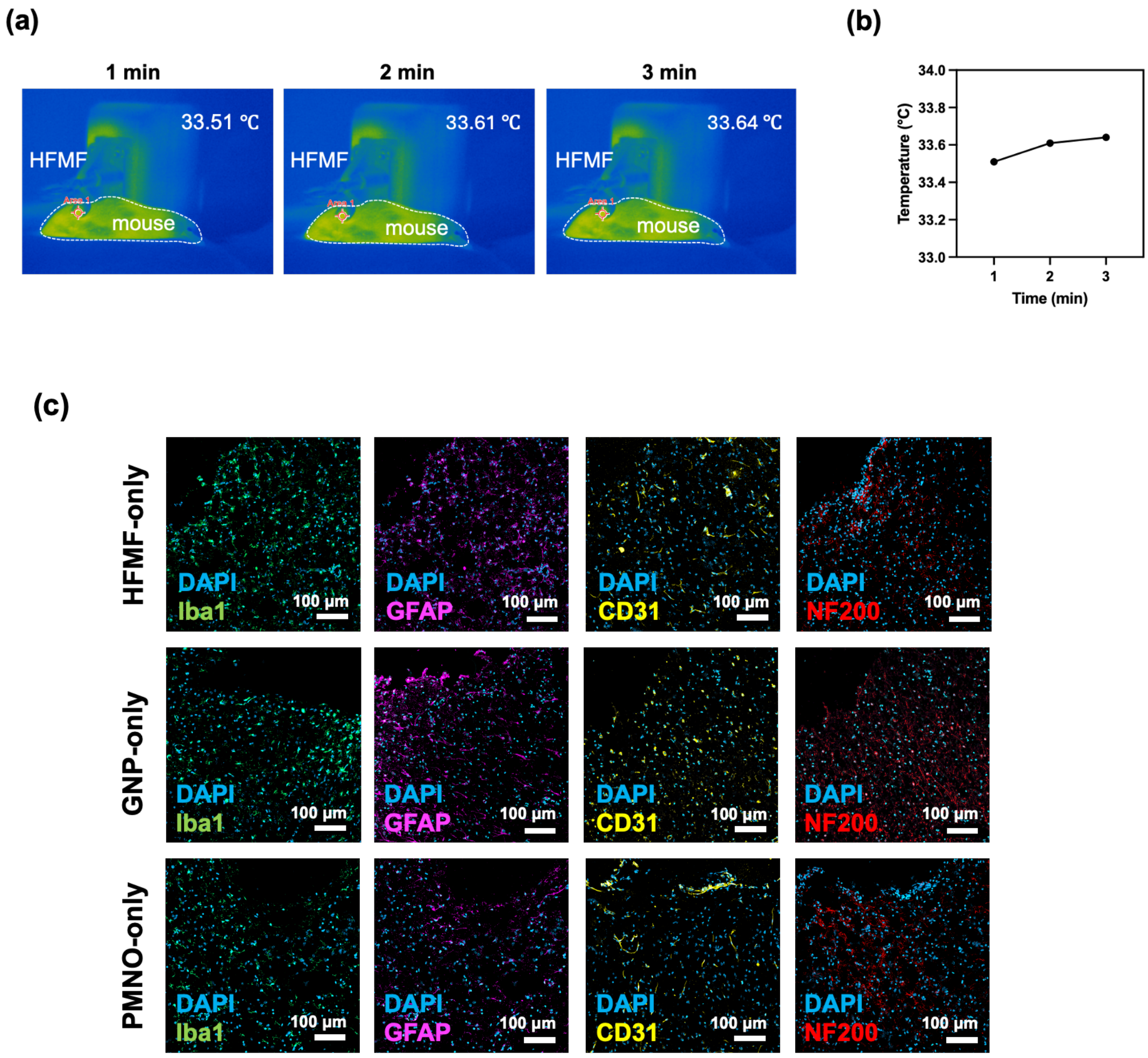

Figure S16. *In vivo* photothermal safety evaluation and short-term peri-lesional pathological responses under HFMF irradiation. (a) Representative real-time infrared thermographic images of mice during a 3 min exposure to high-frequency magnetic field (HFMF) treatment (1 MHz, 2.8 kW). (b) Quantitative localized temperature-time profiles recorded continuously over the 3 min irradiation window. (c) Representative multicolor immunofluorescence micrographs of peri-lesional cortical brain tissue at 7 days post-injury, capturing acute short-term histopathological responses. Confocal sections display reactive astrocytes (purple, GFAP), microglia/macrophages (green, Iba1), endothelial blood vessels (yellow, CD31), and structural neurofilaments (red, NF200) to evaluate reactive gliosis, microglial-mediated neuroinflammation, angiogenesis, and axonal structural integrity, respectively. Cell nuclei are counterstained with DAPI (blue).

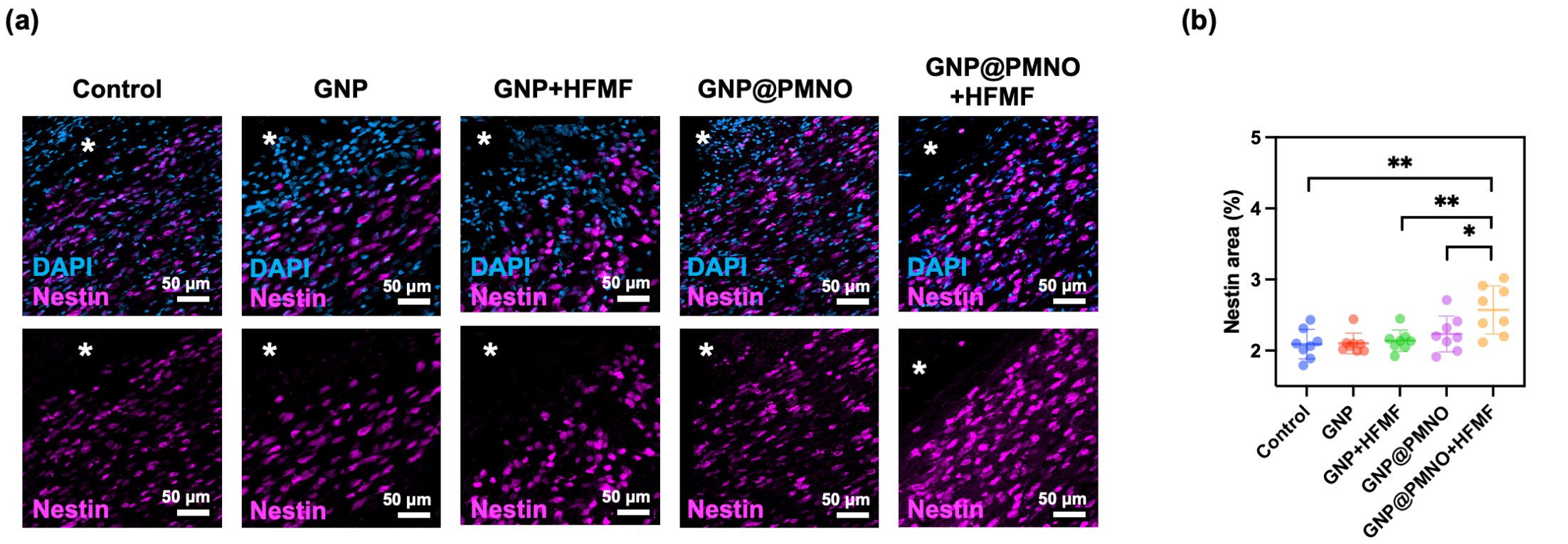

Figure S17. Activation and mobilization of endogenous neural stem cells following TBI. (a) Representative immunofluorescence micrographs showing the recruitment and spatial accumulation of Nestin-positive (Nestin$^+$, purple) undifferentiated endogenous neural stem/progenitor cells adjacent to the impact core (indicated by white asterisks) across experimental cohorts. Cell nuclei are counterstained with DAPI (cyan fluorescence). (b) Corresponding statistical quantification evaluating the relative Nestin expression densities and integrated fluorescence intensities. conditions (n = 8; mean ± s.d.; *p < 0.05, **p < 0.01, one-way ANOVA with Tukey's multiple-comparison test).

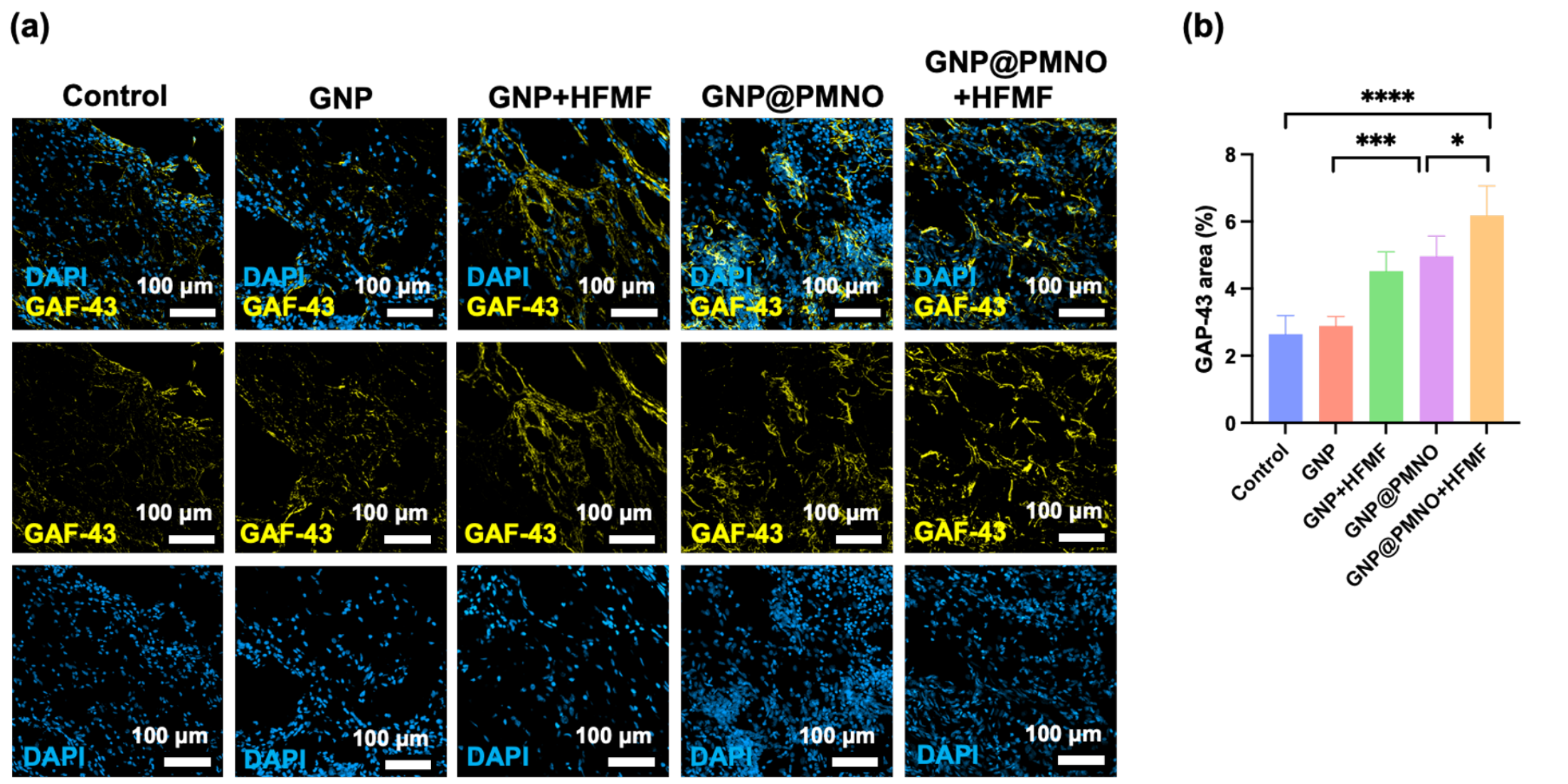

Figure S18. (a) Representative confocal laser scanning microscopy (CLSM) images of peri-lesional brain tissue at 7 days post-injury, illustrating regenerative neuronal activity indicated by growth-associated protein 43-positive (GAP-43$^+$, yellow) immunofluorescence. Nuclei were counterstained with DAPI (blue). (b) Quantitative

analysis of GAP-43-positive cells at the lesion site under different treatment conditions ($n = 5$; mean ± s.d.; *$p < 0.05$, ***$p < 0.001$, ****$p < 0.0001$; one-way ANOVA with Tukey's multiple-comparison test).

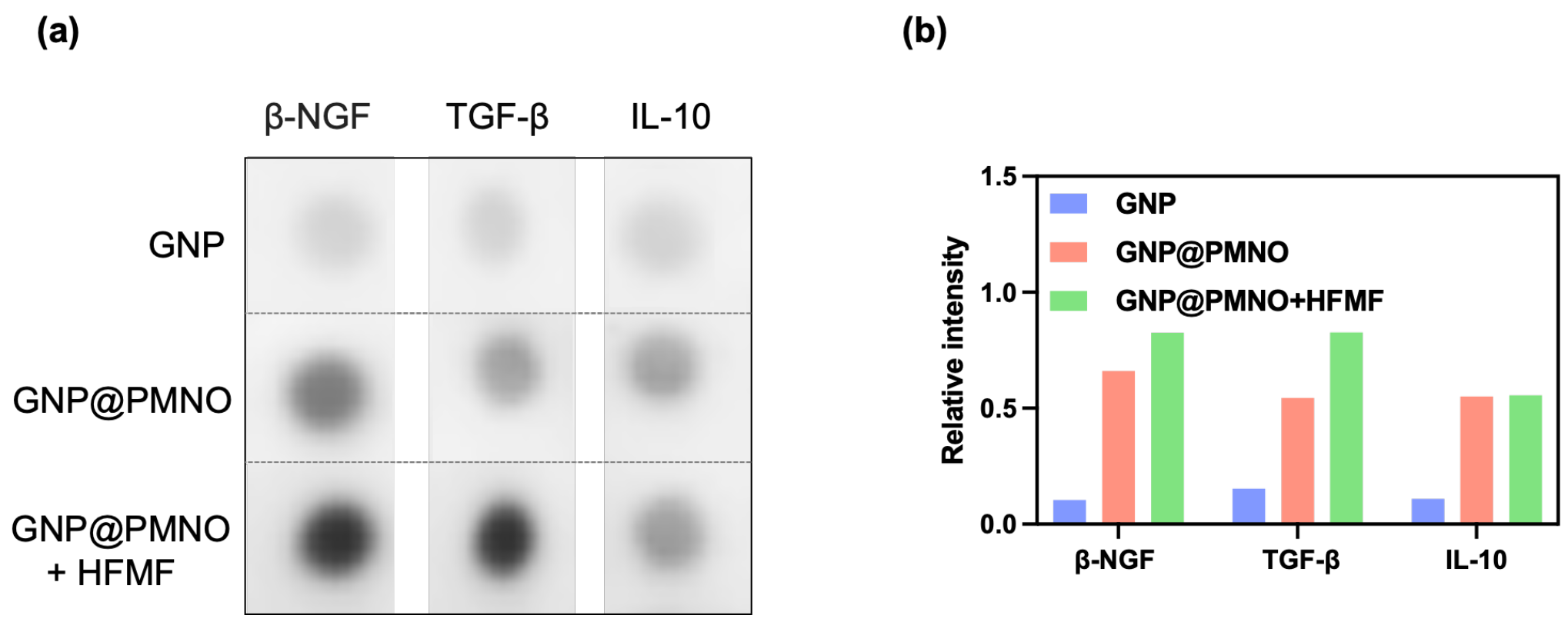

Figure S19. ***In vivo*** **protein microarray analysis elucidating the molecular mechanisms underlying GNP@PMNO-mediated neural regeneration under HFMF stimulation.** (a) Representative protein microarray images showing the expression profiles of neuroregenerative and angiogenesis factors under different treatment conditions. (b) Quantitative analysis of selected cytokines, neurotrophic factors, and angiogenesis-related proteins.

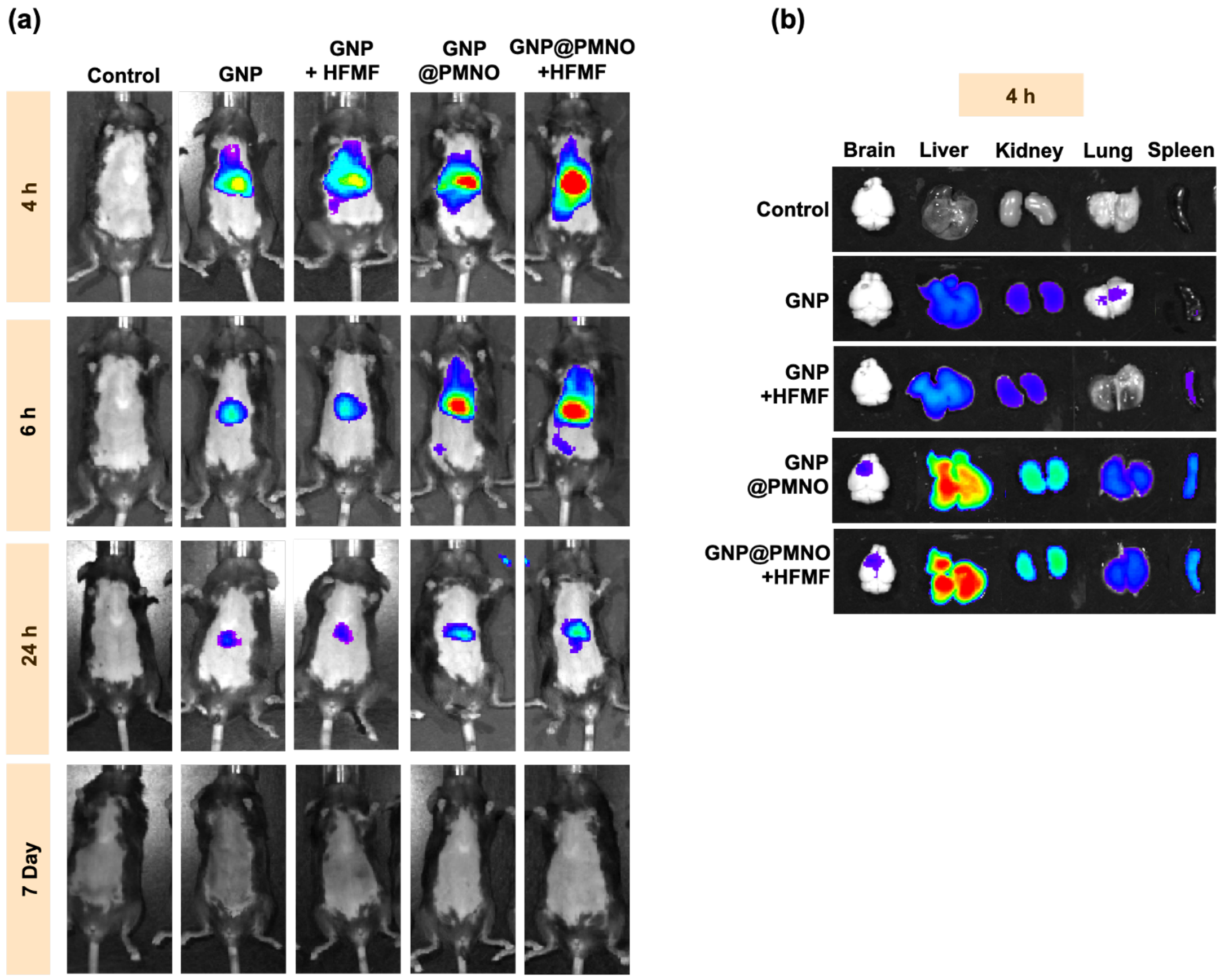

Figure S20. The biodistribution of GNP and GNP@PMNO. (a) *In vivo* imaging system (IVIS) analysis of nanoparticle biodistribution and degradation in the abdominal region at different time points. (b) Ex vivo IVIS imaging of major organs to assess biodistribution in different experimental conditions at 4 h post-injection.

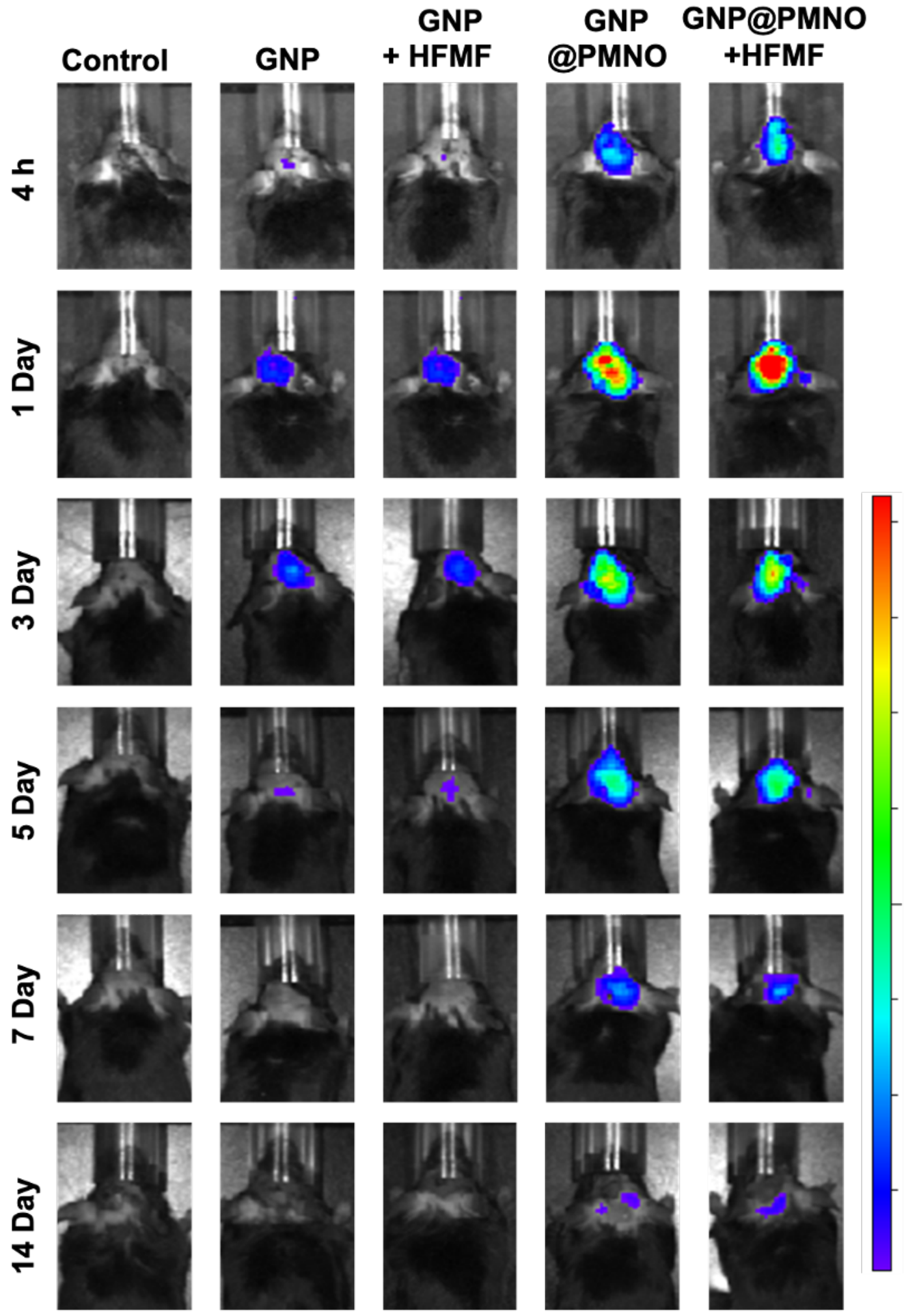

Figure S21. *In vivo* imaging system (IVIS) analysis of nanoparticle accumulation in the brain area at different time points.

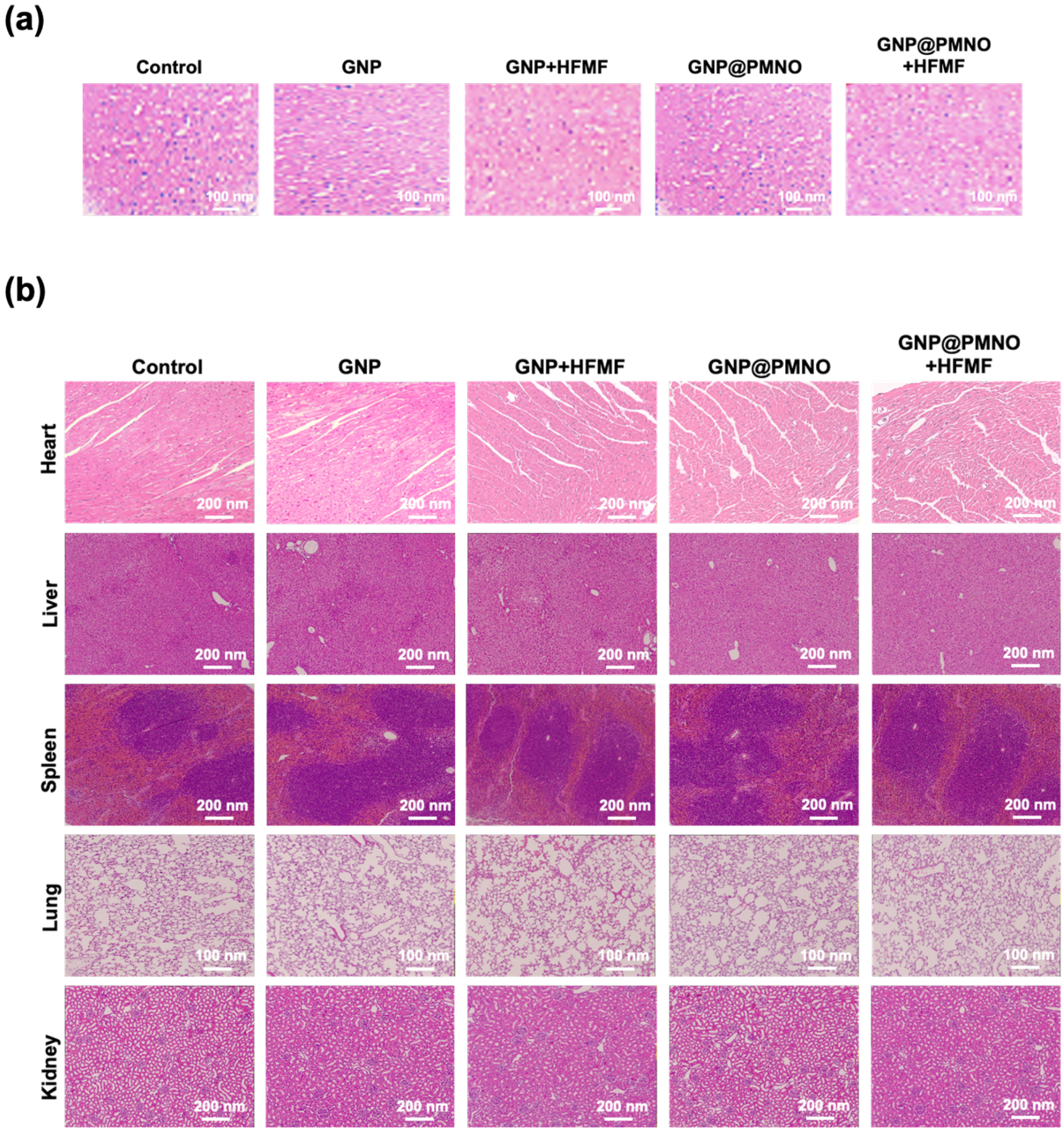

Figure S22. H&E staining of brain (a) and major organs (b) demonstrated the *in vivo* systemic biosafety of different treatments at 28 days post-treatment.

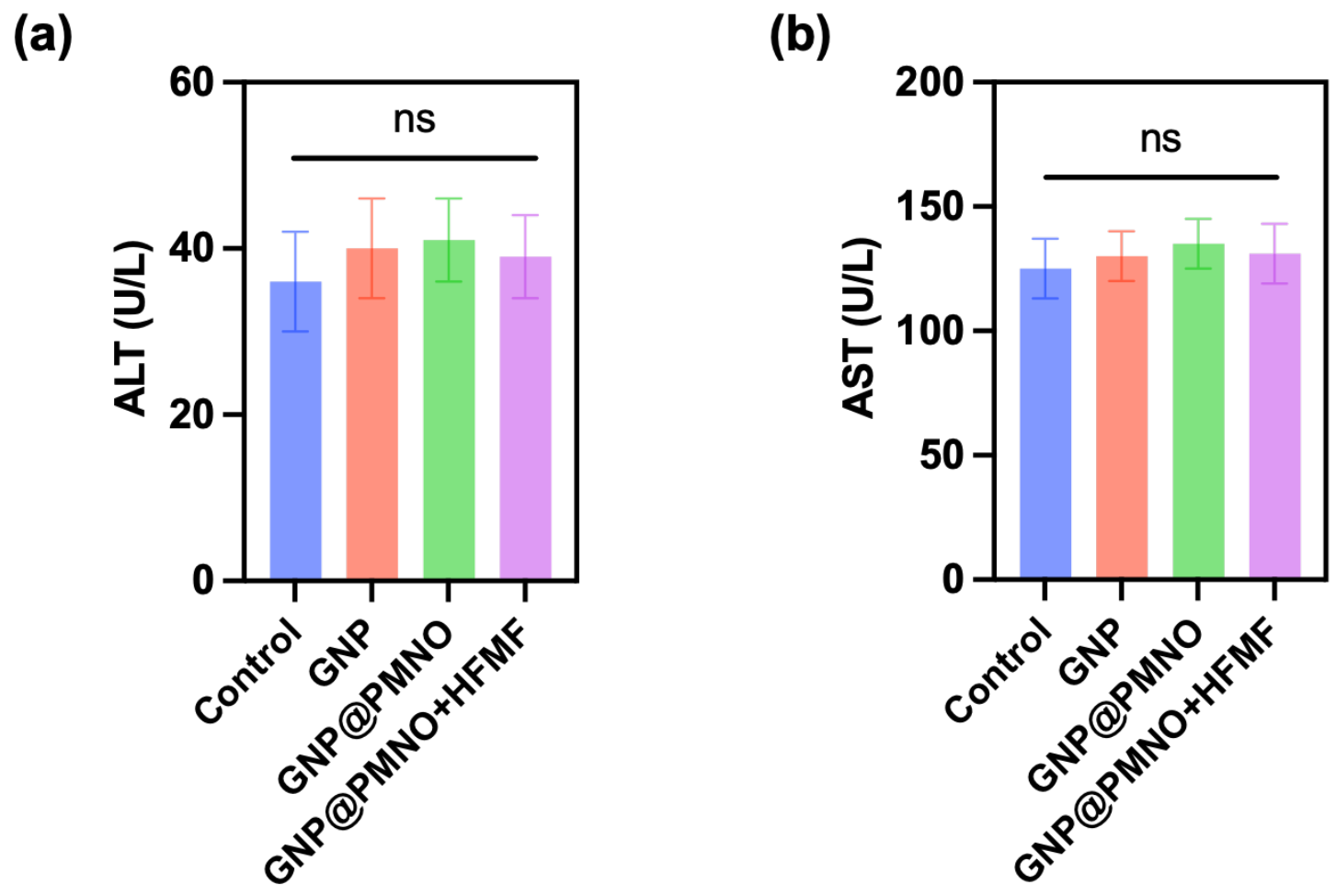

Figure S23. Evaluation of long-term *in vivo* liver toxicity via serum biochemistry analysis. Quantitative measurements of serum hepatic enzyme activities, including (a) alanine aminotransferase (ALT) and (b) aspartate aminotransferase (AST), in mice at 30 days post-treatment across different groups. Data are presented as mean ± SD (n=5). Statistical analysis was performed using one-way ANOVA with Tukey's multiple comparisons test; ns indicates no statistically significant difference ($p>0.05$) among groups.
